# Region‐Specific CD16^+^ Neutrophils Promote Colorectal Cancer Progression by Inhibiting Natural Killer Cells

**DOI:** 10.1002/advs.202403414

**Published:** 2024-05-24

**Authors:** Yan Zhang, Zien Wang, Yu Lu, David J. Sanchez, Jiaojiao Li, Linghao Wang, Xiaoxue Meng, Jianjun Chen, Tran Trung Kien, Ming Zhong, Wei‐Qiang Gao, Xianting Ding

**Affiliations:** ^1^ State Key Laboratory of Systems Medicine for Cancer Renji Hospital School of Biomedical Engineering Shanghai Jiao Tong University Shanghai 200030 China; ^2^ Med‐X Research Institute & School of Biomedical Engineering Shanghai Jiao Tong University Shanghai 200030 China; ^3^ Pharmaceutical Sciences Department College of Pharmacy Western University of Health Sciences 309 East 2nd Street HPC 225 Pomona CA 90025 USA; ^4^ School of Biomedical Engineering Faculty of Engineering and IT University of Technology Sydney Sydney NSW 2007 Australia; ^5^ Department of Gastrointestinal Surgery Renji Hospital School of Medicine Shanghai Jiao Tong University Shanghai 200127 China; ^6^ Oncology department University Medical Shing Mark Hospital 1054 Highway 51, Long Binh Tan Ward, Bien Hoa City Dong Nai 76000 Vietnam; ^7^ State Key Laboratory of Oncogenes and Related Genes Institute for Personalized Medicine School of Biomedical Engineering Shanghai Jiao Tong University Shanghai 200030 China

**Keywords:** cholesterol, CRC, NET, neutrophil, NK cell

## Abstract

The colon is the largest compartment of the immune system, with innate immune cells exposed to antigens in the environment. However, the mechanisms by which the innate immune system is instigated are poorly defined in colorectal cancer (CRC). Here, a population of CD16^+^ neutrophils that specifically accumulate in CRC tumor tissues by imaging mass cytometry (IMC), immune fluorescence, and flow cytometry, which demonstrated pro‐tumor activity by disturbing natural killer (NK) cells are identified. It is found that these CD16^+^ neutrophils possess abnormal cholesterol accumulation due to activation of the CD16/TAK1/NF‐κB axis, which upregulates scavenger receptors for cholesterol intake including CD36 and LRP1. Consequently, these region‐specific CD16^+^ neutrophils not only competitively inhibit cholesterol intake of NK cells, which interrupts NK lipid raft formation and blocks their antitumor signaling but also release neutrophil extracellular traps (NETs) to induce the death of NK cells. Furthermore, CD16‐knockout reverses the pro‐tumor activity of neutrophils and restored NK cell cytotoxicity. Collectively, the findings suggest that CRC region‐specific CD16^+^ neutrophils can be a diagnostic marker and potential therapeutic target for CRC.

## Graphical abstract

A population of CD16^+^ neutrophils that specifically accumulate in colorectal cancer tumor tissues which demonstrates pro‐tumor activity by disturbing nature killer (NK) cells is identified. It is found that these CD16^+^ neutrophils not only competitively inhibit cholesterol intake of NK cells, but also release NETs to induce death of NK cells. CD16‐knockout reverses the pro‐tumor activity of neutrophils and restores NK cell cytotoxicity.

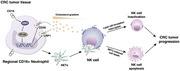

## 1 Introduction

Colorectal cancer (CRC) is a leading cause of death worldwide.^[^ ^1^ ^]^ Immune dysregulation is one of the key contributing mechanisms to CRC progression. The intestines of healthy donors and CRC patients are anatomically and physiologically distinct.^[^ ^2^, ^3^ ^]^ These differences are associated with distinct, region‐specific immune signatures in healthy and CRC patients which may open new avenues for studying CRC pathogenesis and developing therapeutic targets.

The intestinal tract represents the largest compartment of the immune system. The innate immune system in the intestines is continually exposed to antigens and immunomodulatory agents from the diet and the commensal microbiota, as well as a wide range of pathogens including bacteria, fungi, and cancerous cells.^[^ ^4^, ^5^, ^6^ ^]^ As critical effectors of innate immunity, neutrophils have evolved to eliminate diverse pathogens^[^ ^7^ ^]^ and maybe ideally positioned to perform a comparable function in CRC. Interestingly, due to differences in the surrounding tissue microenvironment, tissue source of neutrophils, or their activation status, these cells can produce strikingly different functions.^[^ ^8^, ^9^, ^10^ ^]^

The distinct polarization of neutrophils contributes to their heterogeneous roles in tumor development.^[^ ^11^, ^12^, ^13^ ^]^ In the binary N1/N2 model, N2 neutrophils are described as an immunosuppressive subtype^[^ ^14^ ^]^ and promote tumor development by impairing the antitumor immunity of CD8^+^ T cells^[^ ^15^, ^16^ ^]^ and NK cells.^[^ ^17^ ^]^ Several metabolic shifts are reported to occur in tumor‐associated neutrophils (TANs). For instance, upregulated glycolysis and shift intermediate toward the pentose phosphate pathway (PPP) in neutrophils induce neutrophil extracellular traps (NETs) formation.^[^ ^18^ ^]^ Besides, lipid metabolism is also described as an essential way for TANs to survive under glucose‐limited conditions.^[^ ^19^ ^]^ However, although previous studies have pointed out the correlation between the metabolic features of neutrophils and their functions, few reports revealed the metabolic mechanisms of neutropil immunosuppressive activities particularly in CRC. There is also still a lack of a specific marker, that distinguishes immunosuppressive neutrophils from other neutrophil subtypes in human cancer.

Given the complex roles that neutrophils play in cancer development,^[^ ^20^, ^21^, ^22^, ^23^ ^]^ we sought to identify the specific characteristics of neutrophils in CRC tumor tissues, to elucidate their role in the progression of CRC and identify underlying cellular and molecular mechanisms. Our study identified a population of CD16^+^ neutrophils that specifically accumulated in CRC tumor tissues, which displayed pro‐tumor activity mediated by their inhibition of natural killer (NK) cells. We further found that CD16/TAK1/NF‐κB axis was activated in these CD16^+^ neutrophils, which resulted in the accumulation of cholesterol. This led to competitive inhibition of cholesterol intake by NK cells which interrupted lipid raft formation and therefore diminished NK activation signaling, as well as induced the CD16^+^ neutrophils to release more neutrophil extracellular traps (NETs) that increased NK cell death. Targeting CD16 impaired the competitive cholesterol intake of neutrophils, and restored NK cell cytotoxicity and longevity in CRC.

## 2 Results

### 2.1 CD16^+^ Neutrophils are Accumulated in Tumor Tissues of CRC

**Figure 1:**
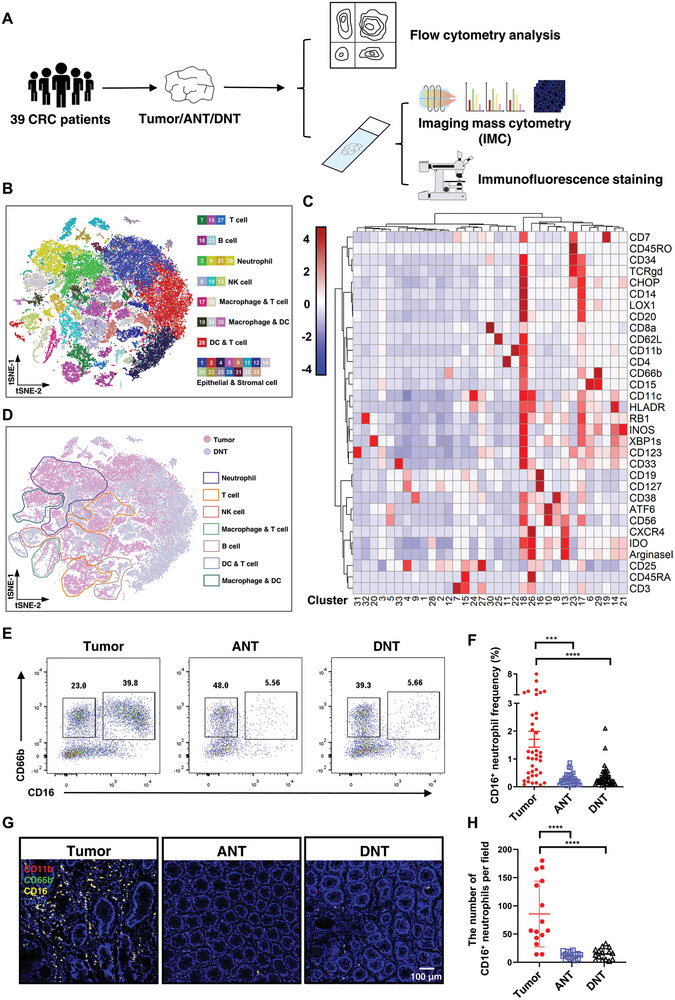
CD16^+^ neutrophils accumulate in CRC tumor tissues. A) Schematic illustration of data acquisition, including sample collection, flow cytometry analysis, imaging mass cytometry (IMC), and immunofluorescence staining. Tumor tissue (tumor), adjacent normal tissue (ANT), and distant normal tissue (DNT) were obtained from 39 CRC patients. B) Combined tSNE plot representing the seven major cell types of CRC and 33 annotated clusters. C) Heat map showing the mean marker expression in each cluster. D) Combined tSNE plot representing different types of immunocytes from tumor tissue (*n* = 10, pink) or DNT (*n* = 10, gray). E) Representative flow cytometry dot plots of CD66b^+^CD16^+^ cell frequencies in tumor, ANT, or DNT. F) Quantification of CD66b^+^CD16^+^ cell frequency in (E) (*n* = 39). G) Representative images of immunofluorescence microscopy of the tumor, ANT, or DNT showing CD11b^+^ (red), CD66b^+^ (green) and CD16^+^ (yellow) cells. H) Quantification of the number of CD66b^+^CD16^+^ cells in (G) (*n* = 15). Mean ± SEM, ***p < 0.001, ****p < 0.0001, by two‐tail *t‐*tests.

To investigate the infiltration of neutrophils in CRC, we collected tumor tissue as well as adjacent normal tissue (ANT) and distant normal tissue (DNT) from the intestines of 39 CRC patients undergoing surgery (**Table** **1**), and analyzed these samples by imaging mass cytometry (IMC), immune fluorescence, and flow cytometry (**Figure** **1**A). First, we used IMC with 33 metal‐labeled antibodies to examine ten CRC tissues and ten DNTs. IMC images were segmented into single cells using a random forest pixel classifier (Ilastik) and CellProfiler, allowing us to identify 52 275 cells in 29 regions of interest (ROI), and all cells were grouped into 33 clusters (Figure 1B). Using the average expression of marker genes in each cluster (Figure 1C), we defined the 33 clusters as seven different cell types, including T cells, B cells, dendritic cells (DC), macrophages, neutrophils, NK cells, as well as epithelial and stromal cells (Figure 1D). Figure 1D showed that CD66b^+^ neutrophils significantly accumulated in CRC tumor tissues.

**Table 1:** Patient information.

Number of patients	Age	Sex	TNM stage	Location	IMC	IF	FC (Granulocytes)	FC (TILs)	MSI/MSS
1	54	M	II	Rectum			T,A,D	T,A,D	MSS
2	49	F	II	Colon	T,D		T,A,D	T,A,D	MSS
3	52	M	II	Rectum		T,A,D	T,A,D		MSS
4	77	F	III	Rectum			T,A,D		MSI
5	67	M	II	Rectum	T,D	T,A,D	T,A,D	T,A,D	MSS
6	56	M	II	Colon			T,A,D	T,A,D	MSS
7	71	F	III	Rectum			T,A,D	T,A,D	MSS
8	52	M	II	Colon		T,A,D	T,A,D		MSS
9	62	F	III	Rectum		T,A,D	T,A,D	T,A,D	MSS
10	62	F	II	Rectum			T,A,D	T,A,D	MSI
11	61	M	II	Colon	T,D		T,A,D	T,A,D	MSS
12	67	M	II	Rectum			T,A,D	T,A,D	MSS
13	54	M	III	Rectum	T,D	T,A,D	T,A,D	T,A,D	MSI
14	54	M	III	Rectum		T,A,D	T,A,D	T,A,D	MSS
15	68	F	II	Colon			T,A,D		MSS
16	43	F	III	Rectum			T,A,D	T,A,D	MSS
17	77	F	II	Rectum	T,D		T,A,D	T,A,D	MSS
18	53	F	II	Rectum			T,A,D		MSS
19	64	M	II	Rectum		T,A,D	T,A,D	T,A,D	MSS
20	64	F	III	Colon		T,A,D	T,A,D	T,A,D	MSI
21	53	M	II	Rectum			T,A,D	T,A,D	MSS
22	57	F	III	Colon			T,A,D	T,A,D	MSS
23	51	F	II	Rectum			T,A,D		MSS
24	67	F	III	Colon		T,A,D	T,A,D	T,A,D	MSI
25	58	F	III	Rectum	T,D		T,A,D	T,A,D	MSS
26	63	M	II	Rectum		T,A,D	T,A,D		MSS
27	59	F	III	Colon			T,A,D	T,A,D	MSI
28	55	M	III	Colon			T,A,D	T,A,D	MSS
29	56	M	II	Rectum		T,A,D	T,A,D	T,A,D	MSS
30	52	F	II	Rectum		T,A,D	T,A,D	T,A,D	MSS
31	76	M	III	Rectum	T,D	T,A,D	T,A,D	T,A,D	MSS
32	66	M	II	Colon			T,A,D	T,A,D	MSS
33	67	F	II	Rectum	T,D	T,A,D	T,A,D	T,A,D	MSS
34	60	M	III	Rectum	T,D		T,A,D	T,A,D	MSS
35	69	M	II	Rectum			T,A,D		MSI
36	50	F	II	Colon			T,A,D	T,A,D	MSS
37	49	M	II	Rectum			T,A,D		MSS
38	55	M	II	Rectum	T,D		T,A,D	T,A,D	MSS
39	71	F	II	Colon		T,A,D	T,A,D	T,A,D	MSS

T, Tumor; A, Adjacent normal tissue; D, Distant normal tissue.

To further confirm the accumulation of neutrophils in CRC, we digested tissues from all 39 samples into single‐cell suspension using lysis buffers, and then stained cells with classical markers of myeloid cells (CD14, CD11b, CD15, CD66b, CD33, and HLA‐DR) and examined them by flow cytometry. We found that CD66b^+^CD11b^+^ neutrophils, especially CD16^+^ neutrophils had significantly greater accumulation in CRC tumors than in ANTs or DNTs (Figure 1E,F). Consistent with this, immunofluorescence staining also showed more CD16^+^ neutrophils accumulating in CRC tumors than in ANTs or DNTs (Figure 1G,H).

Considering that the microsatellite instability of CRC may contribute to the heterogeneity of immunocyte infiltration, we respectively counted the frequencies of CD16^+^ neutrophils in microsatellite stable (MSS) and microsatellite instable (MSI) CRC. CD16^+^ neutrophils were enriched in both MSS and MSI tumors compared to their paired ANTs or DNTs (Figure S1A–D, Supporting Information). Despite microsatellite instability, immunocyte distribution can also vary between tumor core and margin.^[^ ^24^ ^]^ We used hypoxia marker carbonic anhydrase 9 (CAIX/CA9) to specifically label the core region of tumor tissues as previously reported,^[^ ^25^, ^26^ ^]^ and immunofluorescence staining showed that the number of infiltrating CD16^+^ neutrophils were comparable between tumor margin and core (Figure S1E,F, Supporting Information).

Since CD11b and CD66b were also found to be expressed on polymorphonuclear‐myeloid derived suppressive cells (PMN‐MDSC) which were described as a population of CD33^+^HLA‐DR^−^ cells in the tumor microenvironment,^[^ ^27^ ^]^ we then introduced more markers to distinguish these cells with PMN‐MDSC. Flow cytometry shows that these CD66b^+^ neutrophils in CRC had a lower expression of CD33 while they showed a higher level of HLA‐DR compared to peripheral PMN‐MDSC (Figure S1G,H, Supporting Information). These results demonstrated that a large number of CD16^+^ neutrophils but not PMN‐MDSCs were present in CRC tumors compared to in ANTs and DNTs.

### 2.2 NK Cells are Decreased and Disabled in CRC

**Figure 2:**
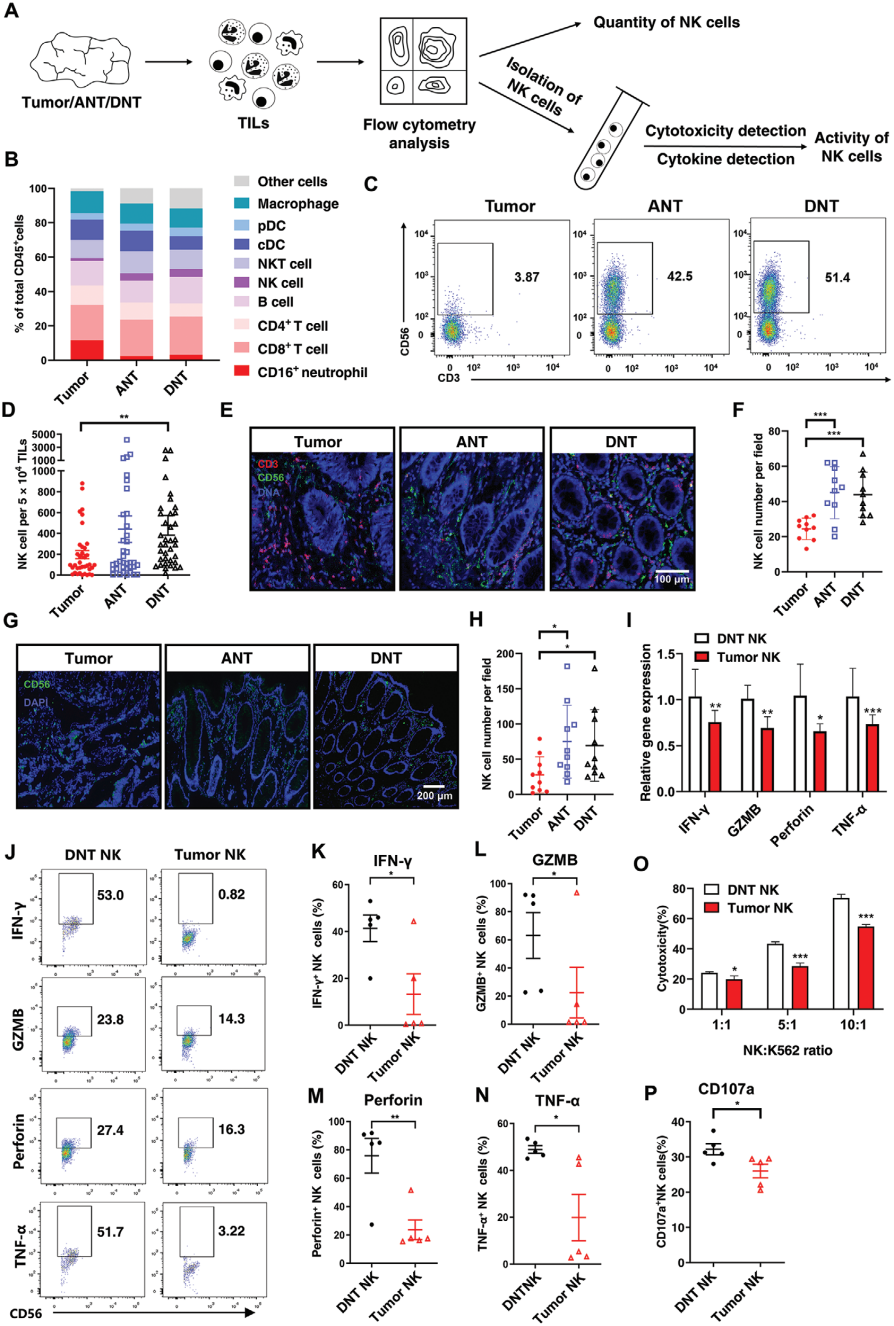
NK cells are decreased and disabled in CRC. A) Schematic illustration of data acquisition, including tumor‐infiltrating lymphocyte (TIL) sorting, flow cytometry analysis, NK cell isolation, NK cell cytokine, and cytotoxicity detection. B) Percentages of B cells, CD4^+^ T cells, CD8^+^ T cells, NK cells, NKT cells, macrophages, cDCs, and pDCs in total CD45^+^ cells analyzed by flow cytometry (*n* = 28). C) Representative dot plot showing percentages of CD3^−^CD56^+^ NK cells in CD3^−^ TILs sorted through magnetic beads sorting, and quantification of the numbers of NK cells from tumor tissues, ANTs, and DNTs. D) Quantification of the number of CD3^−^CD56^+^ NK cells in (C) (*n* = 37). E) Representative images of imaging mass cytometry showing distributions of CD3^+^ (red) and CD56^+^ (green) cells, and the quantification of CD56^+^ NK cells in tumor tissues, ANTs, and DNTs. F) Quantification of the number of NK cells in (E) (*n* = 10). G) Representative images of immunofluorescence showing distributions of CD56^+^ (green) cells, and the quantification of CD56^+^ NK cells in tumor tissues, ANTs, and DNTs. H) Quantification of the number of NK cells in (G) (*n* = 10). I) mRNA expression of antitumor‐related pro‐inflammatory genes (TNF‐α, perforin, GZMB, IFN‐γ) in tumor tissues (tumor NK) and DNT (DNT NK), examined by qPCR. J) Representative dot plots of flow cytometry showing percentages of TNF‐α^+^, perforin^+^, GZMB^+^, and IFN‐γ^+^ tumor NK and DNT NK cells. Both tumor and DNT NK cells were stimulated with IL‐2 (20 ng mL^−1^) and IL‐15 (20 ng mL^−1^) for 12 h before flow cytometry analysis. K) Quantification of the percentages of IFN‐γ^+^, L) GZMB^+^, M) perforin^+^, N) TNF‐α^+^ tumor NK and normal NK cells in (J). O) Quantification of cytotoxicity of tumor NK and normal NK cells. P) Quantification of CD107a^+^ NK cells measured by flow cytometry. Mean ± SEM, *p < 0.05, **p < 0.01, ***p < 0.001, by two‐tail *t*‐tests.

We next investigated whether the infiltration of other CD45^+^ immunocytes in CRC tumors was changed compared to in ANTs and DNTs. CD45^+^ immunocytes were sorted from tumor, ANT, and DNT samples (*n* = 28) followed by flow cytometry analysis and cellular activity detection of specific cells (**Figure** **2**A). We analyzed the frequency of CD8^+^ T cell (CD3^+^CD8^+^CD4^−^), B cell (CD3^−^CD19^+^), CD4^+^ T cell (CD3^+^CD4^+^CD8^−^), NK cell (CD3^−^CD56^+^), NKT cell (CD3^+^TCRVα24Jα18^+^), macrophage (CD11b^+^CD14^+^CD15^−^), conventional dendritic cell (cDC) (CD11b^+^CD11c^+^HLA‐DR^+^), and plasmacytoid dendritic cell (pDC) (CD11b^+^CD123^+^HLA‐DR^−^). There were no significant differences in the average number and activation of infiltrating CD8^+^ T cells among tumor tissues, ANTs, and DNTs (Figure 2B). Immunofluorescence staining also demonstrated that the average number and activation of infiltrating CD8^+^ T cells among tumor tissues, ANTs, and DNTs were comparable, although there was a relatively large intragroup variance (Figure S2A–C, Supporting Information). We also respectively detected CD8^+^ T cell infiltration in MSS and MSI CRC, and the numbers of CD8^+^ T cells were comparable among different types of tissues derived from MSS or MSI CRC (Figure S2D,E, Supporting Information).

The frequency of NK cells (CD3^−^CD56^+^) showed a dramatic decrease in tumor tissues compared to ANTs and DNTs (Figure 2B–D), which aligned with the results of IMC analysis (Figure 1D). To further confirm this finding, IMC was used to visualize the distribution of CD3^−^ (red) CD56^+^ (green) NK cells in frozen tissue slices, which verified that NK cells were not as commonly found in tumor tissues compared to in normal tissues (Figure 2E,F). Consistent with these results, fewer NK cells were detected in CRC tumor tissues by immunofluorescence staining (Figure 2G,H), and there is no difference in this trend between the MSS and MSI CRC (Figure S2F,G, Supporting Information). Furthermore, the numbers of NK cells in the tumor core and margin were comparable (Figure S2H,I, Supporting Information). Innate lymphoid cells (ILC) were previously reported to be a population of CD25^+^CD127^+^ cells in which some subset could also express CD56.^[^ ^28^, ^29^, ^30^ ^]^ To further confirm the CD56^+^ cells that decreased in tumors are NK cells rather than CD56^+^ ILC, we respectively sorted CD56^+^ NK cells and Lin^−^CD25^+^CD127^+^ ILCs by magnetic beads or FACS. qPCR showed that CD56^+^ NK cells rarely expressed CD127 (*IL7R*) and CD25 (*IL2RA*), while CD56 (*NCAM1*) level in ILC was significantly lower than in NK cells (Figure S2J, Supporting Information). Taken together, these findings suggested the number of infiltrating NK cells was decreased in CRC tumor tissues.

The activation and tumor‐killing ability of NK cells rely on their secretion of pro‐inflammatory cytokines such as tumor necrosis factor‐alpha (TNF‐α) and interferon‐γ (IFN‐γ), and cytotoxins such as perforin and GZMB.^[^ ^31^, ^32^ ^]^ To determine whether the antitumor activity of NK cells was also altered in CRC tumor tissues in addition to a reduction in cell number, we separated NK cells from tumor tissues or DNTs by magnetic bead sorting and quantified the mRNA expression levels of TNF‐α, perforin, GZMB and IFN‐γ in NK cells by qPCR after stimulating them with leukocyte activation cocktail. These genes were all significantly downregulated in NK cells isolated from CRC tissues compared to cells isolated from DNTs (Figure 2I). The results were further confirmed by the protein expression level of these genes in NK cells through flow cytometry (Figure 2J–N, Supporting Information). We then investigated whether the cytotoxicity of NK cells isolated from CRC tissues was inhibited. NK cells were co‐cultured with human chronic myeloid leukemia cell line K562 at different ratios (NK: K562, 1:1/1:5/1:10) for 4 h, and cytotoxicity was measured by Promega CytoTox 96 Non‐Radioactive as previously described.^[^ ^33^, ^34^ ^]^ The cytotoxicity of CRC‐derived NK cells on target cells was significantly inhibited compared to NK cells from DNTs (Figure 2O,P, Supporting Information). Collectively, we showed that the number of cells and their cytotoxic activity were both diminished for NK cells isolated from CRC tissues, which suggested the dysfunction of NK cells in CRC tumors.

### 2.3 NK Cells Mediate Pro‐Tumor Activity of CD16^+^ Neutrophils in CRC

**Figure 3:**
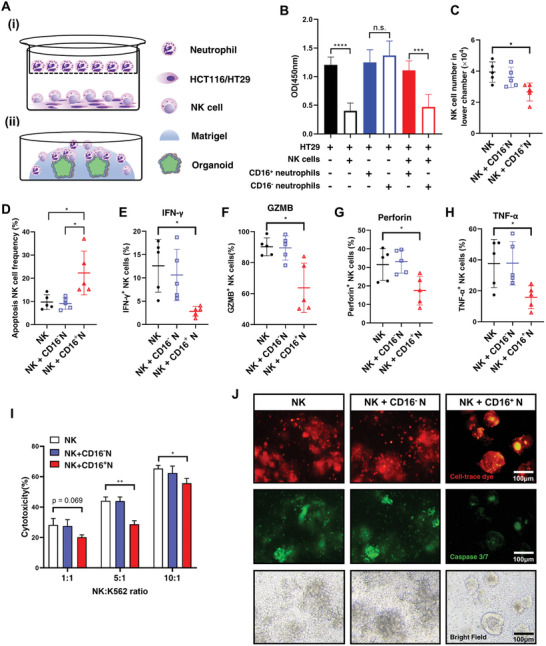
NK cells mediate pro‐tumor activity of CD16^+^ neutrophils in CRC. A) Experimental setup to evaluate in vitro interactions of CD16^+^ neutrophils with NK cells in a transwell co‐culture system (i) and 3D CRC patient‐derived organoid (PDO) co‐culture system (ii). i) After HT29/HCT116 cells were cultured in the lower chamber for 24 h, the sorted NK cells were added to the lower chamber, and the sorted neutrophils were added to the upper chamber, followed by co‐culturing for 48 h. ii) CRC PDOs were first formed in matrigel, and then co‐cultured for 48 h with isolated neutrophils and NK cells added to the 3D culture system. B) Viability of HT29 cells in the co‐cultures as in (Ai) assessed by CCK8 assay (*n* = 9 per group). C) Quantification of the number of NK cells in the lower chamber of the co‐culture system containing HT29 in (Ai). D) Quantification of the frequency of NK cells undergoing apoptosis (Annexin V^+^PI^+^) in the co‐culture system containing HT29. E) Quantification of IFN‐γ^+^, F) GZMB^+^, G) Perforin^+^, and H) TNF‐α^+^ NK cells among NK cells in the co‐culture system containing HT29. I) Quantification of cytotoxicity of NK cells in the co‐culture system containing HT29. J) Representative immunofluorescence images showing PDOs (stained by cell‐trace dye, red) and their apoptosis (stained by caspase 3/7, green). PDOs were co‐cultured with NK cells (NK) alone, NK cells and CD16^−^ neutrophils isolated from DNTs (NK + CD16^−^ Neutrophil), or NK cells and CD16^+^ neutrophils isolated from tumor tissues (NK + CD16^+^ Neutrophil). Mean ± SEM, *p < 0.05, **p < 0.01, ***p < 0.001, ****p < 0.0001, by two‐tail *t‐*tests.

To evaluate how CD16^+^ neutrophils and NK cells were involved in the development of CRC, we built an in vitro co‐culture system comprising CD16^+^ neutrophils sorted from CRC tissues or CD16^−^ neutrophils from DNTs by FACS in the upper chamber, and NK cells sorted from peripheral blood of healthy donors (HD) as well as CRC MSS cell line HT29 or MSI cell line HCT116 in the lower chamber (**Figure** **3**A(i)). In the absence of NK cells in the co‐culture system, neither CD16^+^ neutrophils nor CD16^−^ neutrophils showed direct pro‐tumor activity on HT29 (Figure 3B). When CD16^+^ neutrophils and NK cells were both added to the co‐culture system with HT29, the cytotoxicity of NK cells toward HT29 was significantly reduced to a level similar to HT29 without NK cells. In contrast, CD16^−^ neutrophils did not affect the cytotoxic effects of NK cells on HT29 (Figure 3B). The same trend was also observed in the co‐culture system in which MSI cell line HCT116 was involved (Figure S3A, Supporting Information). These results indicated that CD16^+^ neutrophils could block the antitumor activity of NK cells to promote CRC development.

We then explored whether NK cell was involved in the pro‐tumor activity of CD16^+^ neutrophils since NK cells in CRC tumor tissues had lower cell numbers and a dysfunctional state. During co‐culture with HT29 or HCT116, the existence of CD16^+^ neutrophils significantly reduced the number of NK cells (Figure 3C; Figure S3B, Supporting Information) and enhanced NK cell apoptosis (Figure 3D; Figure S3C,E, Supporting Information), suggesting that induced apoptosis might be contributing to the low number of NK cells in CRC. NK cells co‐cultured with CD16^+^ neutrophils also showed significantly lower expression of TNF‐α, perforin, GZMB, and IFN‐γ (Figure 3E–H; Figure S3F–K, Supporting Information), and these results were confirmed by qPCR (Figure S3L,M, Supporting Information). Besides, CD16^+^ neutrophils apparently reduced the cytotoxicity of NK cells on target cells (Figure 3I; Figure S3N–P, Supporting Information). We further confirmed these results using a different co‐culture model of CRC patient‐derived organoids (PDOs) co‐cultured with neutrophils and NK cells (Figure 3A(ii)). Fluorescence staining showed a high number of apoptotic PDOs (Caspase 3/7^+^, green) when they were co‐cultured with NK cells, or NK cells together with CD16^−^ neutrophils (Figure 3J; Figure S3Q, Supporting Information). In contrast, apoptotic PDOs were significantly reduced after CD16^+^ neutrophils were added to the co‐culture system (Figure 3J; Figure S3Q, Supporting Information), suggesting that CD16^+^ neutrophils could diminish the cytotoxicity of NK cells. Collectively, our findings indicated that CD16^+^ neutrophils might modulate tumor‐infiltrating NK cells by inhibiting their cytotoxicity and enhancing apoptosis, thereby promoting CRC tumor development.

### 2.4 CD16 Enhanced Cholesterol Uptake in Neutrophils Through CD16/TAK1/NF‐κB Axis

**Figure 4:**
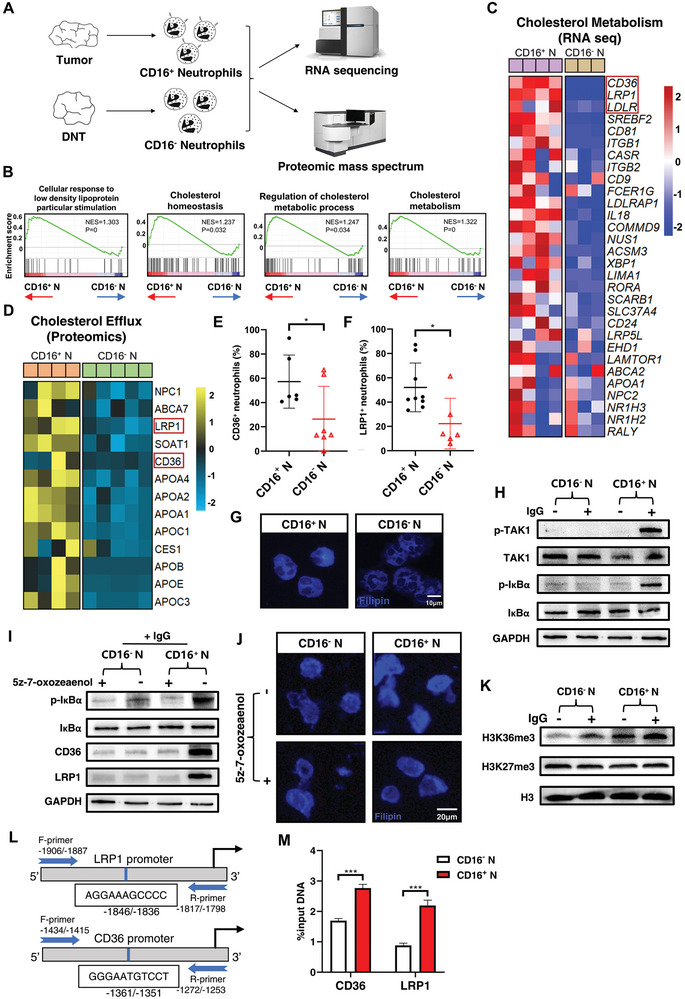
Cholesterol is accumulated in CRC regional CD16^+^ neutrophils. A) Schematic illustration of data acquisition, including neutrophil isolation, RNA sequencing, and protein mass spectrum. B) GSEA enrichment analysis of RNA‐seq data from CD16^+^ neutrophils in CRC tumor tissues and CD16^−^ neutrophils in DNT. C) Heatmap showing differential gene expression in CD16^+^ neutrophils and CD16^−^ neutrophils from RNA‐seq, genes ranked by *p*‐value. D) Heatmap showing the expression of core proteins within the cholesterol efflux pathway in CD16^+^ neutrophils and CD16^−^ neutrophils from the proteomic mass spectrum. E) Quantification of the frequency of CD36^+^ and F) LRP1^+^ cells in CD16^+^ and CD16^−^ neutrophils. G) Filipin III staining of CD16^+^ and CD16^−^ neutrophils. Mean ± SEM, *p < 0.05, **p < 0.01, by two‐tail *t*‐tests. H) Western blot of TAK1 and IκBα phosphorylation in CD16^−^ and CD16^+^ neutrophils with or without IgG (1 µg mL^−1^) stimulation. I) Western blot of IκBα phosphorylation, CD36, and LRP1 in CD16^−^ and CD16^+^ neutrophils with or without 5z‐7‐oxozeaenol (20 nm) followed by IgG stimulation. J) Filipin III staining of CD16^−^ and CD16^+^ neutrophils cultured with or without 5z‐7‐oxozeaenol (20 nm) followed by IgG stimulation. K) Western blot of H3K27me3 and H3K36me3 in CD16^−^ and CD16^+^ neutrophils with or without IgG (1 µg mL^−1^) stimulation. L) Schematic diagram of human LRP1 and CD36 promoter showing predicted NF‐κB binding site in the regulatory region. The upstream regions are numbered in relation to the transcription initiation site. Primers were designed to involve the NF‐κB binding site sequence in the PCR product. M) ChIP assays of the H3K36me3 enrichment around the NF‐κB binding site of CD36 and LRP1 in IgG‐stimulated neutrophils. Mean ± SEM, *p < 0.05, **p < 0.01, ***p < 0.001, by two‐tail *t‐*tests.

To elucidate the mechanism by which neutrophils exert an immunosuppressive effect on NK cells in CRC, we isolated CD16^+^ neutrophils from CRC tumor tissues or CD16^−^ neutrophils from DNTs by FACS and analyzed them using RNA‐seq and proteomic mass spectrum (**Figure** **4**A). Using Gene Set Enrichment Analysis (GSEA), we identified several enriched pathways associated with lipoprotein and cholesterol metabolism in CD16^+^ neutrophils (Figure 4B). Of note, a majority of genes involved in cholesterol metabolism were significantly upregulated in CD16^+^ neutrophils (Figure 4C). Among these, the most significantly enriched genes were related to cholesterol uptake, such as CD36^[^ ^35^, ^36^ ^]^ and lipoprotein receptor‐related protein‐1 (LRP1),^[^ ^37^ ^]^ and low‐density lipoprotein receptor (LDLR).^[^ ^38^ ^]^ Proteomic mass spectrum analysis also showed a significant upregulation of core proteins involved in the cholesterol efflux pathway in tumor‐derived neutrophils, including CD36 and LRP1 (Figure 4D; Figure S4A, Supporting Information), which matched the RNA‐seq data. These results were confirmed by flow cytometry, where the expression levels of CD36, LRP1, and LDLR were all increased in CD16^+^ neutrophils compared to CD16^−^ neutrophils (Figure 4E,F; Figure S4B–D, Supporting Information).

Since CD36, LRP1, and LDLR induce cellular uptake of cholesterol and play an important role in cellular cholesterol homeostasis,^[^ ^39^ ^]^ we next examined the amount of cholesterol in CD16^+^ and CD16^−^ neutrophils. The CD16^+^ neutrophils showed significantly higher intracellular cholesterol levels compared to CD16^−^ neutrophils (Figure S4E, Supporting Information). The same finding was confirmed by quantitative detection and staining for filipin III, a cholesterol probe (Figure 4G; Figure S4F, Supporting Information). These results suggested that CD16^+^ neutrophils in CRC tumor tissues had stronger lipid metabolism, especially cholesterol uptake, and maintained a higher level of intracellular cholesterol.

CD16 activation has been shown to be a potent inducer of signal transduction and consequent cell phenotype modulation.^[^ ^40^ ^]^ Specifically, it has been reported that Transforming Growth Factor β Activated Kinase 1 (TAK1) plays a crucial role in the CD16‐induced signaling cascade by being phosphorylated upon stimulation, and subsequently activating several transcriptional factors including NF‐κB.^[^ ^41^, ^42^ ^]^ We examined whether CD16 could enhance cholesterol uptake in neutrophils by triggering the TAK1/NF‐κB axis. We obtained CD16^+^ and CD16^−^ neutrophils from CRC patients and stimulated them with IgG, the ligand of CD16. We observed an increase in TAK1 and IκBα phosphorylation in CD16^+^ neutrophils (Figure 4H), which was effectively abolished by treatment with the TAK1 inhibitor 5z‐7‐oxozeaenol (Figure 4I). 5z‐7‐oxozeaenol also inhibited expression of CD36 and LRP1 (Figure 4I; Figure S4G, Supporting Information), and consequently downregulated the intracellular cholesterol level in CD16 neutrophils (Figure 4J; Figure S4H, Supporting Information).

Given NF‐κB's capacity to participate in epigenetic modulation via histone methylation,^[^ ^43^, ^44^ ^]^ we then investigated whether the TAK1/NF‐κB axis activation regulated cholesterol uptake by histone modifications in CD16^+^ neutrophils. Our findings revealed that stimulated CD16^+^ neutrophils demonstrated significantly higher levels of H3K36me3 than CD16^−^ neutrophils, while levels of H3K27me3 were comparable between the two groups (Figure 4K). We then investigated whether the upregulation of H3K36me3 enhanced the transcriptional activity of NF‐κB on CD36 and LRP1. The binding sites of p50 on the promoters of CD36 and LRP1 were predicted on FIMO, and then we designed primers specific for them for chromatin‐immunoprecipitation‐qPCR (ChIP‐qPCR) to determine the enrichment of H3K36me3 around the p50 binding sites (Figure 4L). Notably, the enrichment of H3K36me3 on the promoters of LRP1 and CD36 significantly increased in CD16^+^ neutrophils compared to CD16‐ neutrophils (Figure 4M). Taken together, CD16/TAK1/NF‐κB axis co‐operated with histone modification to transcriptionally enhance cholesterol uptake of CD16^+^ neutrophils in CRC.

### 2.5 CD16^+^ Neutrophils Disable NK Cells by Interrupting Lipid Raft Formation

**Figure 5:**
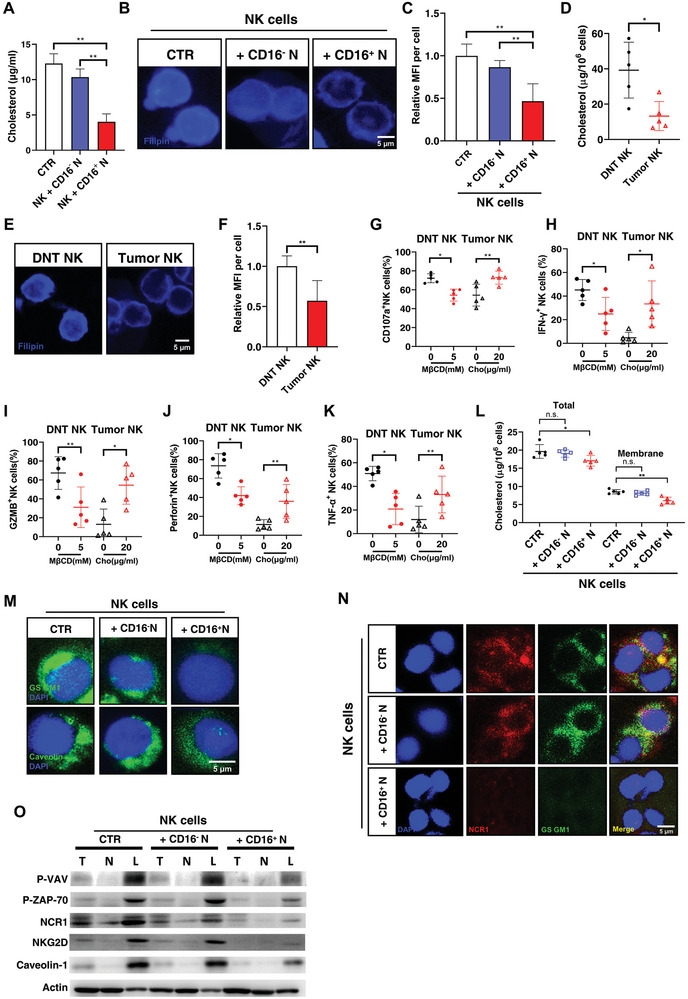
CD16^+^ neutrophils disable NK cells by interrupting lipid raft formation. A) Cholesterol quantification of the medium in the co‐culture system of HCT 116 and NK cells with none (CTR), CD16^−^ neutrophils (CD16^−^ N) and CD16^+^ neutrophils (CD16^+^ N). B) Filipin III staining of NK cells alone (NK) and co‐cultured with CD16^−^ neutrophils or CD16^+^ neutrophils. C) Quantification of fluorescence intensity in (B). D) Cholesterol quantification of isolated normal tissue‐infiltrating CD56^+^ NK cells (DNT NK) or tumor‐infiltrating CD56^+^ NK cells (Tumor NK). E) Filipin III staining of normal tissue‐infiltrating CD56^+^ NK cells (DNT NK) or tumor‐infiltrating CD56^+^ NK cells (Tumor NK). F) Quantification of fluorescence intensity in (E). G) Quantification assessed by flow cytometry of CD107a^+^ cells in DNT NK cells cultured in complete RPMI 1640 medium with 0/5 mm MβCD, and Tumor NK cells with 0/20 exogenous cholesterol (µg mL^−1^) followed by cocktail stimulation. H) The frequency of IFN‐γ^+^, I) GZMB^+^, J) Perforin^+^, and K) TNF‐α^+^ cells in DNT NK cells cultured in complete RPMI 1640 medium with 0/5 mm MβCD, and Tumor NK cells with 0/20 exogenous cholesterol (µg mL^−1^) followed by cocktail stimulation. L) Cholesterol quantification of total cell lysate (Total, left) or plasma membrane (Membrane, right) from NK cells alone (CTR), or co‐cultured with CD16^−^ neutrophils (+ CD16^−^ N) or CD16^+^ neutrophils (+ CD16^+^ N). M) Representative images of immunofluorescence showing distributions of ganglioside GM1 (GS GM1) and caveolin‐1 in NK cells alone (CTR) and co‐cultured with CD16^−^ neutrophils (+ CD16^−^ N) or CD16^+^ neutrophils (+ CD16^+^ N). N) Representative images of immunofluorescence showing distributions of NCR1 (red) and GS GM1 (blue) in NK cells alone (CTR) and co‐cultured with CD16^−^ neutrophils (+ CD16^−^ N) or CD16^+^ neutrophils (+ CD16^+^ N). NK cells were sorted from the co‐culture system by magnetic sorting before immunofluorescence staining. O) The levels of indicated proteins in total cell lysate (T), nonlipid raft (N), or lipid raft fraction (L) of NK cells alone (CTR) or co‐cultured with CD16^−^ neutrophils (+ CD16^−^ N) or CD16^+^ neutrophils (+ CD16^+^ N). Mean ± SEM, *p < 0.05, **p < 0.01, by two‐tail *t*‐tests.

After finding that CD16^+^ neutrophils had stronger cholesterol uptake than CD16^−^ neutrophils, we then explored whether the extracellular cholesterol level was also dysregulated in CRC microenvironment. We first determined the extracellular cholesterol concentration in the co‐culture system with HCT116, NK cells, and neutrophils as shown in Figure 3A(i). We found that CD16^+^ neutrophils significantly reduced the cholesterol level in the medium compared to CD16^−^ neutrophils (**Figure** **5**A). Then we wondered if the cholesterol concentration in vitro could reflect that in microenvironment in vivo. We examined the cholesterol concentration in tumor extracellular fluid (EF) and serum, which showed that the cholesterol level in EF was much closer to that in culture media rather than serum (Figure S5A, Supporting Information). Meanwhile, the intracellular cholesterol level of NK cells decreased after co‐culturing with CD16^+^ neutrophils compared to CD16^−^ neutrophils (Figure 5B,C). Co‐culturing with NK cells did not affect the intracellular cholesterol level of CD16^+^ neutrophils (Figure S5B,C, Supporting Information). We then found that NK cells in tumor tissues had lower intracellular cholesterol than cells in DNTs (Figure 5D). This result was further confirmed by Filipin staining (Figure 5E,F). These results suggested an inhibition of cholesterol intake in NK cells from CRC tumor tissues, which may be related to competitive cholesterol uptake by CRC CD16^+^ neutrophils.

We then asked if CD16^+^ neutrophils induced NK cell dysfunction by the inhibition of cholesterol intake of NK cells. To explore the correlation between lower cholesterol levels and dysfunction of NK cells, exogenous cholesterol or methyl‐β‐cyclodextrin (MβCD), an inhibitor for cholesterol intake, were respectively added to the cell medium of NK cells from tumor tissues or from DNTs to modulate the intracellular cholesterol level of NK cells (Figure S5D, Supporting Information). MβCD showed an inhibition to NK cell cytotoxicity similar to NK cells co‐cultured with CD16^+^ neutrophils, while higher cholesterol levels significantly enhanced the cytotoxicity of NK cells (Figure 5G–K; and Figure S5E, Supporting Information). Meanwhile, exogenous cholesterol had no effects on the apoptosis frequency of NK cells (Figure S5F, Supporting Information). Then we detected CD107a expression in NK cells under different cholesterol levels and found that higher cholesterol levels contributed to the NK cell CD107a expression with cholesterol concentration among 10–100 µg mL^−1^ which is similar to tissue EF (Figure S5G, Supporting Information) while NK cell activation no longer enhanced by the increase of cholesterol concentration when it came to the serum concentration (≈1000–2000 µg mL^−1^; Figure S5G, Supporting Information). In conclusion, lowering the cholesterol amount in NK cells induced by CD16^+^ neutrophils could result in the reduction of NK cell cytotoxicity.

Cholesterol is an important component of the plasma membrane, which plays a vital role in membrane trafficking and signaling.^[^ ^45^ ^]^ We found that CD16^+^ neutrophils significantly decreased the membrane cholesterol level of NK cells in the co‐culture system (Figure 5L), which indicated that the decreased intracellular cholesterol level could influence NK cell activation by affecting plasma membrane signal transduction. Cholesterol is mainly enriched in lipid rafts in the plasma membrane.^[^ ^46^ ^]^ Fluorescence staining revealed that decreased expression of lipid raft markers (ganglioside GM1 and caveolin‐1) was observed in NK cells co‐cultured with CD16^+^ neutrophils, compared to other groups (Figure 5M; Figure S5H,I, Supporting Information). Lipid rafts organize and determine the function of multiprotein complexes involved in several aspects of signal transduction, thus regulating cell homeostasis and activation in immune cells.^[^ ^47^ ^]^ Next, we detected the expression and localization of natural cytotoxicity triggering receptor 1 (NCR1), a receptor engaged in NK cell activation.^[^ ^48^ ^]^ NK cells co‐cultured with CD16^+^ neutrophils exhibited significantly low expression of NCR1 on the cell membrane along with decreased GS GM1, compared to the other groups (Figure 5N; Figure S5J,K, Supporting Information). Consistently, the results of western blotting showed that the level of NCR1 and natural killer group 2 member D (NKG2D), another major receptor in NK cell activation,^[^ ^49^ ^]^ significantly decreased in lipid raft fraction (L), but not total cell lysate (T) or nonlipid raft (N), of NK cells co‐cultured with CD16^+^ neutrophils, compared to the other two groups (Figure 5O). Together, these results demonstrated that the low intracellular cholesterol level in NK cells caused by CD16^+^ neutrophils mainly affected NK cell cytotoxicity by weakening lipid raft formation, which blocked receptor signaling.

### 2.6 CD16^+^ Neutrophil‐Derived NETs Induce NK Cell Death

**Figure 6:**
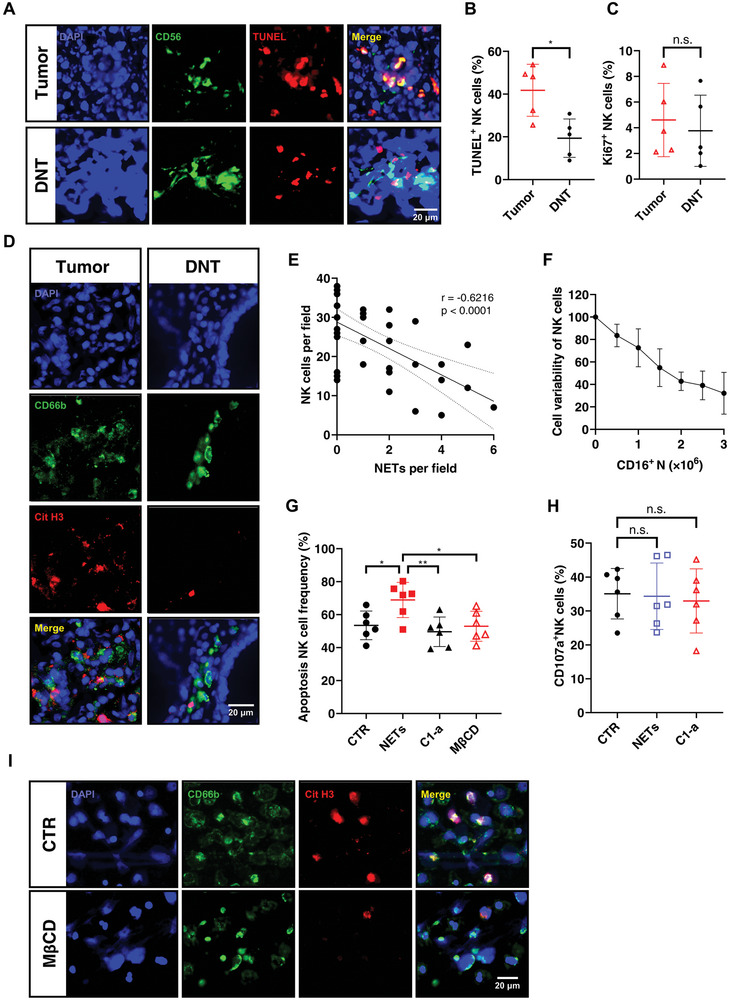
CD16^+^ neutrophil‐derived NETs induce NK cell death in CRC. A) Representative images of immunofluorescence microscopy of CRC tumor tissue and DNT showing CD56^+^ cells (green) and TUNEL (TdT‐mediated dUTP Nick‐End Labeling, red). B) Quantification of dead NK cells as TUNEL^+^CD56^+^ cells in tumor tissues and DNT. C) Quantification of proliferative NK cells as Ki67^+^CD56^+^ cells in tumor tissues and DNT. D) Representative images of immunofluorescence microscopy of tumor tissues and DNT showing CD66b^+^ cells (green) and Cit H3 (red). E) Pearson correlation between NET and NK cell (CD56^+^) in both tumor tissues and DNT (*n* = 31). F) Percentage of viable NK cells after exposure to PMA‐induced NETs isolated from the indicated number of CD16^+^ neutrophils. G) Quantification of the frequency of NK cells undergoing apoptosis (Annexin V^+^PI^+^) after exposure to PMA‐induced NETs isolated from CD16^+^ neutrophils, in the presence of an inhibitor for NET release (C1‐a) or an inhibitor for cholesterol intake (MβCD). H) Percentage of CD107^+^ NK cells after exposure to PMA‐induced NETs isolated from CD16^+^ neutrophils, in the presence of an inhibitor for NET release (C1‐a). I) Representative images of immunofluorescence microscopy of CD16^+^ neutrophils with or without 5 mm MβCD showing CD66b^+^ cells (green) and Cit H3 (red). Mean ± SEM, *p < 0.05, **p < 0.01, by two‐tail *t*‐tests.

We sought to elucidate the mechanism by which CD16^+^ neutrophils decreased the number of NK cells in CRC tumor tissues. We found that alterations in the number of NK cells in CRC tumor tissue were associated with increased NK death (TdT‐mediated dUTP Nick‐End Labeling, TUNEL^+^), not with changes in cell proliferation (**Figure** **6**A–C; Figure S6A, Supporting Information). Previous study has shown that neutrophil extracellular traps (NETs) released from neutrophils could induce cell death in chronic inflammation,^[^ ^50^ ^]^ so we then investigated whether NETs play an important role in apoptosis of NK cells induced by CD16^+^ neutrophils. Notably, trap–DNA structures, such as citrullinated H3 (cit H3) and myeloperoxidase (MPO), representing classical NETs were observed only in tumor tissues rather than DNTs, and these NETs were found to be primarily of neutrophil origin (Figure 6D; Figure S6B–D, Supporting Information). Consistent with this, the amounts of NETs in CRC tumor tissue were inversely correlated with NK cell number (Figure 6E).

To investigate the direct inhibitory effects of NETs on NK cells, NK cells were incubated with NETs isolated from different numbers of phorbol myristate acetate (PMA)‐induced CD16^+^ neutrophils. The viability of NK cells was found to gradually decrease with higher amounts of NETs in an approximately linear manner (Figure 6F). Consistent with this, NK cells showed significantly higher apoptosis when incubated with NETs isolated from 2.0 × 10^6^ neutrophils treated with PMA (Figure 6G). Pharmacological blockage of NET release (Cl‐amidine treatment) resulted in decreased death of NK cells (Figure 6G), but the activity of NK cells was not affected by NETs (Figure 6H). In addition, we found that inhibition of cholesterol intake in CD16^+^ neutrophils by MβCD could significantly reduce their release of NETs (Figure 6I, Figure S6E, Supporting Information) and subsequently alleviate CD16^+^ neutrophil‐induced NK cell death (Figure 6G). These findings collectively point to a dysregulated mechanism in CRC development, whereby cholesterol accumulation in CD16^+^ neutrophils not only inhibits the activity of NK cells by interrupting lipid raft formation and blocking antitumor signaling pathways but also induces NK cell death by release of NETs.

### 2.7 CD16‐Knockout in Neutrophils Relieved the Immunosuppression in CRC

**Figure 7:**
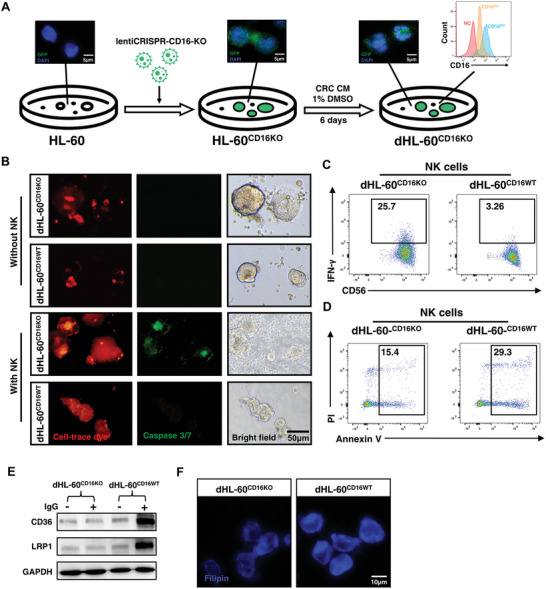
CD16‐knockout in neutrophils relieved the immunosuppression in CRC. A) The schematic diagram shows the establishment of dHL‐60^CD16KO^. HL‐60 was cultured with lentiCRISPR‐CD16‐KO for 48 h, and purified with puromycin (2 µg mL^−1^) for 6 days to obtain HL‐60^CD16KO^. Then we induced HL‐60^CD16KO^ into dHL‐60^CD16KO^ with HCT116 conditional medium and 1% DMSO. The transfected and knockout efficiency was confirmed by immunofluorescence and flow cytometry. B) Representative immunofluorescence images showing PDOs (stained by cell‐trace dye, red) and their apoptosis (stained by caspase 3/7, green). PDO was co‐cultured with dHL‐60^CD16KO^ or dHL‐60^CD16WT^ with or without the presence of peripheral NK cells. C, D) Representative dot plots of flow cytometry showing percentages of C) IFN‐γ^+^ NK cells and D) apoptotic NK cells (Annexin V^+^PI^+^) in the co‐culture system described in (B). NK cells were isolated from the co‐culture system by magnetic sorting before flow cytometry analysis. E) Western blot of CD36 and LRP1 of dHL‐60^CD16KO^ and dHL‐60^CD16WT^ with or without IgG stimulation. F) Filipin III staining of dHL‐60^CD16KO^ and dHL‐60^CD16WT^. Mean ± SEM, *p < 0.05, by two‐tail *t*‐tests.

To investigate whether CD16‐blockage can weaken the pro‐tumor activity of CD16^+^ neutrophils, we first established a CD16‐knockout HL‐60 (HL‐60^CD16KO^), a human promyelocyte cell line,^[^ ^51^ ^]^ through CRISPR‐Cas9, and induced the HL‐60^CD16KO^ into differentiated HL‐60^CD16KO^ (dHL‐60^CD16KO^) cells via tumor conditioned medium with 1% DMSO (**Figure** **7**A). Next, we established the PDO‐NK‐dHL‐60 co‐culture system as described above (Figure 3A). After 48 h, we observed that PDOs co‐cultured with dHL‐60^CD16KO^ and NK cells presented a significant increase of Caspase 3/7 signal compared to those with dHL‐60^CD16WT^. These results indicated that dHL‐60^CD16KO^ lost the immunosuppressive activation on NK cells and led to an increase in the apoptosis of PDOs within the co‐culture system (Figure 7B; Figure S7A,B, Supporting Information). dHL‐60^CD16KO^ or dHL‐60^CD16WT^ alone had no direct cytotoxicity to PDO which was similar to CD16^+^ and CD16^−^ neutrophils (Figure 7B; Figure S7A,B, Supporting Information). To exclude the killing effect of NK cells on dHL‐60^CD16KO^ which may also result in the loss of dHL‐60^CD16KO^’s immunosuppressive activation, we examined the viability of dHL‐60^CD16KO^ or dHL‐60^CD16WT^ by CCK8 after co‐cultured with stimulated NK cells and PDOs, and found that the existence of NK cells did not influence the viability of dHL‐60 (Figure S7C, Supporting Information). Notably, IFN‐γ expression of NK cells co‐cultured with dHL‐60^CD16KO^ cells significantly increased compared to co‐cultured with dHL‐60^CD16WT^ (Figure 7C; Figure S7D, Supporting Information). CD16‐knockout in dHL‐60 also abolished NK cell apoptosis induced by neutrophils (Figure 7D; Figure S7E, Supporting Information). Furthermore, dHL‐60^CD16KO^ showed a significantly lower level of CD36 and LRP1 expression, which might result in the inhibition of cholesterol uptake by dHL‐60 (Figure 7E). Filipin staining confirmed the downregulation of intracellular cholesterol levels in CD16^KO^ dHL‐60 compared to CD16^WT^ dHL‐60 (Figure 7F; Figure S7F, Supporting Information). These results suggest that CD16‐knockout serves to alleviate the pro‐tumor activity of neutrophils by impairing CD36/LRP1‐induced cholesterol uptake and subsequently restoring NK cell cytotoxicity and longevity.

## 3 Discussion

The tumor microenvironment in CRC is complicated and modulated by many cellular and molecular factors,^[^ ^2^, ^52^ ^]^ such as immunocytes, cytokines, and metabolic intermediates. However, the nature of these factors and the mechanisms by which they regulate CRC progression is not completely understood. For instance, it is unclear whether there are specific immunocyte populations that exist in CRC tissue with the ability to affect tumor progression. In this study, we identified a population of CD16^+^ neutrophils, which were found to specifically accumulate in CRC tumor tissues and play an important role in helping tumor growth. Further, we uncovered that the pro‐tumor activity of this neutrophil population is mediated by their abnormal cholesterol accumulation which consequently induced the inhibition and apoptosis of NK cells. Specifically, we elucidated their potential mechanisms of action in promoting CRC tumor growth by 1) competitive inhibition of NK cell cholesterol intake to interrupt lipid raft formation and block NK cell antitumor signaling pathways, and 2) releasing more NETs to induce death of NK cells. These findings pointed to the possibility of employing region‐specific CD16^+^ neutrophils as a future diagnostic marker or therapeutic target for CRC.

The most interesting finding in our study is that pro‐tumor activity in CRC was only conferred by CD16^+^ but not CD16^−^ neutrophils. CD16 belongs to a group of membrane proteins, where the constant regions of immunoglobulin heavy chains are ligands of receptors that are designated as Fc receptors (FcR), and FcR that specifically bind IgG antibodies are termed FcγR.^[^ ^53^ ^]^ Previous studies have shown that FcγRs are expressed on mature myeloid and lymphoid cells, and Fcγ receptor III (CD16) is selectively expressed on mature but not immature granulocytes.^[^ ^54^ ^]^ Our findings are supported by other reports indicating tumor infiltration by myeloid cells in CRC.^[^ ^55^, ^56^ ^]^ However, while some groups identified myeloid cells as CD16^+^ macrophages,^[^ ^55^ ^]^ others named them as regular neutrophils (CD66b^+^).^[^ ^56^ ^]^ These discrepancies might have arisen due to overlapping or missing myeloid cell markers used in the experiments. In our study, we applied multiple channel flow cytometry, immunofluorescence staining, and imaging mass cytometry to clearly demonstrate that the myeloid cells infiltrating into CRC tumor tissues were CD16‐expressing neutrophils (CD66b^+^CD16^+^CD33^−^HLA‐DR^+^ neutrophils). Furthermore, CD16 was found to possess signal transduction function and could influence the phenotype of macrophages and neutrophils through its downstream signaling pathway.^[^ ^41^, ^57^ ^]^ In this study, we found that the activation of the CD16/TAK1/NF‐κB axis in CD16^+^ neutrophils is indispensable for the immunosuppressive role of neutrophils in CRC. Meanwhile, CD16 blockage in neutrophils can remove the pro‐tumor activity of CD16^+^ neutrophils, which indicates that CD16 was not only a diagnosis marker for CRC but also a potential immunotherapy target.

Our results showed that CD16^+^ neutrophils have pro‐tumor activities that require the involvement of NK cells, and these activities were reversed in co‐culture systems lacking NK cells, indicating a crucial role of NK cells rather than other immune cell subsets in CD16^+^ neutrophil‐mediated CRC progression. Multiple epidemiological studies have revealed conflicting evidence on the effects of tumor infiltration by neutrophils, found to be associated with both improved and worsened survival in CRC patients.^[^ ^13^, ^23^, ^58^, ^59^, ^60^ ^]^ Others have shown that the interplay between neutrophils and CD8^+^ T cells may improve the survival rate in human CRC.^[^ ^61^, ^62^ ^]^ The apparently heterogeneous activities of neutrophils in CRC progression might at least in part be because the observations in previous experimental studies were conducted at different stages of CRC since neutrophil infiltration was shown to be a favorable prognostic factor only in the early stages of colon cancer.^[^ ^61^ ^]^ Our results suggested that CD16 expression on neutrophils might be an indicator of the transition of function between neutrophil subtypes. Adding to the findings from other studies that also observed interactions between neutrophils and NK cells in tumor progression,^[^ ^63^, ^64^ ^]^ our study found a specific population of CD16^+^ neutrophils that interacts with NK cells in a region‐specific manner in CRC. We found that NK cells were the most changed immune cell subset in CRC tumors, which mediated the pro‐tumor activities of CD16^+^ neutrophils. These findings give further evidence to the possibility that different populations of neutrophils perform different functions in various tumor types and microenvironments. Whether the same neutrophil population exists in other tumor types, and if so whether they exhibit the same pro‐tumor activities and interactions with NK cells warrants further investigation.

Our study pointed to the interesting mechanism of cholesterol metabolism in the interactions of CD16^+^ neutrophils with NK cells, adding to the other known NK cell‐related inhibitory mechanisms in cancer.^[^ ^65^, ^66^ ^]^ In CRC, we found that cholesterol is accumulated in CD16^+^ neutrophils through the upregulation of CD36 and LRP1, which we believe could influence the tumor microenvironment in one of two ways. First, decreased cholesterol levels in tumor tissues with CD16^+^ neutrophils might affect the function of NK cells. Our work suggests that CD16^+^ neutrophils can inhibit the activity of NK cells by interrupting lipid raft formation and blocking antitumor signaling pathways. There have been several researches focusing on the relationship of cholesterol and NK cell activity which gave contradictory conclusions in vitro or in the peripheral blood.^[^ ^67^, ^68^ ^]^ This divergence may have resulted from the very different cholesterol concentrations in different microenvironments. In our work, we found that appropriately increasing cholesterol concentration in a tumor environment, which is generally in a state of cholesterol deficiency, contributes to tumor‐infiltrating NK cell activation. However, a high level of cholesterol in serum which is much higher than that in tissue EF has no positive effect on the peripheral NK cells. Meanwhile, our results also showed that cholesterol accumulation in CD16^+^ neutrophils could induce higher NET release, which results in the apoptosis of NK cells in CRC. NETs are structures of chromatin filaments coated with histones, proteases, and granular and cytosolic proteins, which neutrophils release to attach to and eliminate target cells.^[^ ^69^ ^]^ Recent evidence suggests that cholesterol crystals activate the inflammasome through the CD36 receptor, which mediates the release of NETs in neutrophils,^[^ ^70^ ^]^ consistent with our observations in this study. Collectively, these new mechanisms by which CD16^+^ neutrophils inhibit the activity of NK cells and promote their death may play an important role in CRC.

In conclusion, our study revealed that a population of region‐specific CD16^+^ neutrophils was present in CRC tumor tissues, and displayed pro‐tumor activity by inhibiting NK cells. These findings present a new link between cholesterol uptake dysregulation and the immune inhibitory activity of CD16^+^ neutrophils in CRC, as well as define new mechanisms by which CD16^+^ neutrophils interfere with the antitumor activity of NK cells, which have important implications for future diagnostic and therapeutic studies in clinical investigations of CRC.

## 4 Experimental Section

### Human Specimens

The study was approved by the Research Ethics Committee of Renji Hospital, Shanghai Jiao Tong University School of Medicine. (Approval No. LY2023‐268‐B). No patients received radiotherapy or chemotherapy before surgery. Tissue slices were obtained from patients diagnosed with CRC by clinical pathological examination, who presented to the Department of Gastrointestinal Surgery, Renji Hospital (Shanghai, China) between January 2018 and September 2022. Samples were obtained before capecitabine adjuvant treatment. Peripheral venous blood of healthy donors was collected at Renji Hospital. The pathological information of 39 patients enrolled in this study was retrieved from the Pathology Department of Renji Hospital. All patients provided written informed consent before enrolment.

### Statistical Analysis

All analyses in this study were performed using IBM SPSS STATISTICS 22.0 software and GraphPad Prism 8 software. Statistical analysis was performed using the two‐tailed Student's *t*‐test, and results were presented as mean ± SEM unless otherwise indicated. Differences were considered statistically significant if *p* < 0.05.

## Conflict of Interest

The authors declare no conflict of interest.

## Author Contributions

Y.Z., Z.W., and Y.L. contributed equally to this work. Y.Z., W.Q.G., and X.T.D. conceived and supervised the project. Y.Z., Z.E.W., Y.L., L.H.W., and X.X.M. designed and performed the research. Y.L. and M.Z. collected and provided the CRC samples. J.J.C., T.T.K., Z.E.W., and Y.L. performed the data analyses. Z.E.W., Y.L., L.H.W., and X.X.M. performed experiments. Y.Z., M.Z., W.Q.G., and X.T.D. interpreted the results. Y.Z., Z.E.W., D.J.S, J.J.L, J.J.C., W.Q.G., and X.T.D. wrote the paper with input from all the other authors.

## Supporting information

Supporting Information

Supporting Information

## Data Availability

The data that support the findings of this study are available from the corresponding author upon reasonable request.
