# Supplementary material for: Region‐Specific CD16+ Neutrophils Promote Colorectal Cancer Progression by Inhibiting Natural Killer Cells
Source: Adv Sci (Weinh). 2024 May 24;11(29):2403414. doi: 10.1002/advs.202403414 (PMC11304263; doi:10.1002/advs.202403414)
Supplement: Supplementary file 1 — Supporting Information [file ADVS-11-2403414-s002.docx]

**Supporting information:**

Supplemental Methods and Materials

Figure S1 to S7

Table S1

**Supplementary materials and methods**

**Imaging mass cytometry analysis**

Imaging mass cytometry (Fluidigm, Hyperion) was used to scan the tissue sections to generate multiplexed images. To segment image data into single-cell data, we used CellProfiler software (the Whitehead Institute for Biomedical Research and MIT’s CSAIL) to obtain mask files, which were fed into histoCAT software for tSNE and PhenoGraph analyses and cell neighborhood analysis.

**CRC Patient-derived organoid (PDO)**

CRC tumor tissues were dissected into 1-2 mm^2^ fragments and treated with pre-warmed digestive solution (2 mg ml^-1^ collagenase type IV, 0.2 mg ml^-1^ hyaluronidase, 0.2 mg ml^-1^ DNase I, 10 μM Y-27632, Sigma) at 37 ℃ for 30 min. After incubation, fetal calf serum (Gibco) was added and the mixture was put over a 100 μm cell strainer to remove large fragments. Then cells were subsequently spun at 1,000 rpm for 3 min. The pellet was resuspended in basal culture medium (Advanced DMEM/F12 with 1× GlutaMAX-1, 1× HEPES, 10 mM nicotinamide, 1× B27, 1 mM N-acetylcysteine, 10nM gastrin) and spun again at 1,000 rpm for 3min. The procedure was repeated twice to remove debris and collagenase. After that, tumor cells were resuspended in Matrigel (Corning) and plated into 48-well plates (40 μl Matrigel per well), and 250 μl growth medium (basal culture medium plus 500 ng ml^-1^ recombinant human R-Spondin 1, 50 ng ml^-1^ recombinant human EGF, 100 ng ml^-1^ recombinant human Noggin, 500 nM A-86-01 and 3 μM SB202190) was added into every well.

For organoid culture, organoid growth medium was refreshed every 2 days. To passage the organoids, Matrigel was broken up by pipetting and organoids were collected in a tube. The organoids were centrifuged at 1,000 rpm for 3 min and the medium removed. Triple Express (Invitrogen, 5 ml) was added and the organoids were incubated at 37 ℃ for approximately 5 min. Every minute, a visual check was done to verify the size or the organoids. Care was taken not to treat the organoids to long with Triple Express. Then FCS and medium were added and cells were spun down at 1,500 rpm for 3 min. After that, the pellet was taken up in Matrigel and cells were plated in droplets of 5–10 ml each. After allowing the Matrigel to solidify, HICS (for normal organoids) or HICS minus Wnt (for tumoroids), both supplemented with LY27632 (10 μM), was added to the plates and organoids were incubated at 37 ℃.

**Cell sorting**

For the magnetic sorting of CD56^+^ NK cells from CRC tumor tissues (Tumor NK) and DNT (DNT NK), CRC tumor and DNT were dissected into 1-2 mm^2^ fragments and treated with pre-warmed digestive solution (2 mg ml^-1^ collagenase type IV, 0.2 mg ml^-1^ hyaluronidase, 0.2 mg ml^-1^ DNase I, Sigma) at 37 ℃ for 30min, and the single cell suspensions were treated with BD Pharm Lyse lysing solution for red blood cell lysing. After washing 3 times with PBS, NK cells were counted and resuspended in PBS (10^7^ cells in per 80 μl). Then 20 μl microbeads (Miltenyi Biotec) were added into per 80 μl cell suspension and the mixture was incubated at 4 ℃ for 15 min. Labeled cell suspension was then applied onto the magnetic column. After washing the column to remove unlabeled cells, the column was removed from the magnetic separator, and the labeled cells were immediately flushed out and collected. For the magnetic sorting of CD66b^+^ neutrophils in the co-culture system, the suspended cells in the system were sucked out and counted, and then magnetic sorting protocol was proceeded.

For the FACS enrichment of NK cells derived from peripheral blood of healthy donors which were used for the co-culturing with other cells, as well as PMN-MDSC derived from peripheral blood of CRC patients, fresh anticoagulant whole blood was diluted with equal volume PBS, and human lymphocyte separation medium was added into the diluted blood. After 500g centrifugating for 30 min, immunocyte population was sucked out and then the CD45^+^CD11b^-^CD3^-^CD19^-^CD56^+^ NK cells or the CD45^+^CD11b^+^CD66b^+^ PMN-MDSC in immunocyte population were enriched by FACS.

For the FACS enrichment of ILC and CD16^+^ neutrophils derived from CRC tumor tissues, as well as CD16^-^ neutrophils derived from distant normal tissues (DNT), CRC tumor and DNT were dissected and digested into single cell suspension. After red blood cell lysing and washing with PBS, the Lin^-^CD25^+^CD127^+^ (Lin: CD3, CD11b, CD19, CD14, CD66b, CD16) ILC and CD45^+^CD11b^+^CD66b^+^CD16^+/-^ neutrophils in single cell suspension were enriched by FACS.

**Co-culture**

For the co-culture of CRC PDO and isolated neutrophils and NK cells, peripheral NK cells were firstly activated with IL-2 (20 ng ml^-1^) and IL-15 (20 ng ml^-1^) (leukocyte activation cocktail) for 12 h, and then 10^5^ NK cells and 10^5^ neutrophils were resuspended with organoid growth medium (200 μl) plus IL-2 (20 ng ml^-1^) and IL-15 (20 ng ml^-1^). The co-culture system was incubated at 37 ℃ for 48 h before subsequent experiments.

For the co-culture of HT29/HCT116 and isolated neutrophils and NK cells, 2.5×10^5^ HT29 or HCT116 were sed into the lower chamber of a 24-well culture plates with matching Transwells (0.4 μm pore size, Corning) and kept in 500μl McCoy’s 5A medium (Gibco) for 24 h. Then 2×10^5^ NK cells were added to the lower chamber after activated with IL-2 (20 ng ml^-1^) and IL-15 (20 ng ml^-1^) (leukocyte activation cocktail) for 12 h and 2×10^5^ neutrophils were added to the upper chamber while replacing complete DMEM with complete RPMI-1640. The co-culture system was incubated at 37 ℃ for 48 h before subsequent experiments.

**Proteomics**

Proteins were precipitated with chloroform and methanol after cells were lysed in RIPA buffer (Sigma) and protease inhibitor (Roche). Proteins were digested with trypsin (Promega) and the resulting peptides were purified on reverse phase material (Thermo Fisher).

The LC-MS instrumentation consisted of an EASY-nLC 1200 system (Thermo Fisher Scientific) and an Orbitrap mass spectrometer (Q Exactive HF-X, Thermo Fisher Scientific). Peptides were separated on a trap column (Acclaim PepMapTM 100, 75 μm × 2 cm, C18, 3 μm, 100 Å) at 2 µL min^-1^ with solvent A (0.1% (v/v) formic acid), and then eluted with a 120 min gradient on an analytical column (Acclaim PepMapTM RSLC, 75 μm × 25 cm, C18, 2 μm, 100 Å) at a flow rate of 300 nL min^-1^. The gradient elution program was: 0-1 min, 1% to 8% solvent B (0.1% (v/v) formic acid and 80% (v/v) acetonitrile); 1-80 min, 8% to 22% solvent B; 80-98 min, 22% to 28% solvent B; 98-112 min, 28% to 36% solvent B; 112-116 min, 36%-100% solvent B; 116-120 min, 100% solvent B.

**CD16-knockout in HL-60**

HL-60 cell line was purchased from Pricella, Wuhan. The sgRNA sequences for FCGR3B (coding CD16) or nontargeting control were obtained from addgene library (https://www.addgene.org/pooled-library/zhang-human-gecko-v2/) and are listed in **Table S1**. Lentivirus used for CD16-knockout and non-targeting-control were constructed by AZENTA. Briefly, psPAX2 and pMD2.G plasmids were co-transfected into 293T cells. The supernatant containing the virus was collected after viral packaging for 72 hours, and the virus-containing supernatant was obtained by filtration to obtain the crude virus. The crude virus was then concentrated by ultrafiltration or precipitated with PEG 8000 to obtain a purified virus. The purified lentiCRISPR-CD16-KO virus and lenti-CRISPR-CD16-WT were further obtained by high-speed centrifugation.

For CD16-knockout in HL-60, HL-60 cells were sed into 24 well plate (2×10^5^ per well). After 3 hours, lentiCRISPR-CD16-KO or lentiCRISPR-CD16-WT were added into HL-60 with MOI as 100. Reinfection with the virus 24 hours later. The virus medium was replaced with normal RPMI1640 medium containing puromycin (2 μg ml^-1^) 48 hours after the first transfection. HL-60^CD16KO^ cells and HL-60^CD16WT^ were collected 5 days later, and the transfection and knockout efficiency was confirmed by fluorescence microscopy and flow cytometry.

**Analysis of microsatellite instability status**

Microsatellite instability status was analyzed by immunohistochemical (IHC). MMR proteins including MLH1, MSH2, MSH6, and PMS2 were detected with IHC in Renji hospital. Loss of an MMR protein was defined as the absence of nuclear staining of tumor cells in the presence of positive nuclear staining in internal controls. Tumor tissues with loss of at least one MMR protein were defined as deficient MMR (dMMR) or microsatellite instable (MSI), and tumors with intact MMR protein expression designated as proficient MMR (pMMR) or microsatellite stable (MSS).

**Flow cytometry analysis**

To determine the frequency of different immunocyte subsets, cells (10^6^ cells per tube) were stained by monoclonal antibodies BB515 Rat anti-CD11b (BD Biosciences), BV650 mouse anti-human CD16 (BD Biosciences), PE Mouse anti-human CD66b (BD Biosciences), PerCP-Cy5.5 mouse anti-human CD15 (BD Biosciences), Horizon V500 mouse anti-human CD14 (BD Biosciences), BV421 mouse anti-human CD33 (BD Biosciences), APC mouse anti-human CD34 (BD Biosciences), BV421 mouse anti-human CD117 (BD Biosciences), PE mouse anti-human CD38 (BD Biosciences), APC-H7 mouse anti-human HLA-DR (BD Biosciences), BV421 mouse anti-human CD19 (BD Biosciences), APC or APC-Cy7 mouse anti-human CD3 (BD Biosciences), PE-Cy7 mouse anti-human CD4 (BD Biosciences), APC or PE mouse anti-human CD8 (BD Biosciences), PE mouse anti-human Vα24Jα18 (BD Biosciences), APC mouse anti-human CD11c (BD Biosciences), APC mouse anti-human CD56 (BD Biosciences), PE mouse anti-human CD107a (BD Biosciences), IFN-γ mouse anti-human BV421 (BD Biosciences), FITC mouse anti-human perforin (BD Biosciences), V450 mouse anti-human CD36 (BD Biosciences), BVB510 mouse anti-human LRP1 (BD Biosciences), V450 mouse anti-human CD33 (BD Biosciences), or APC-Cy7 mouse anti-human HLA-DR (BD Biosciences) at 4 °C for 30 min. Flow cytometry was performed on an LSRFortessa (BD Biosciences) and analyzed using FlowJo Version X (Tree Star, Inc).

**Immunofluorescence staining**

Frozen slides were thawed at room temperature, blocked with 10% serum (specific to the species in which the secondary antibodies were raised) in PBS and stained overnight at 4°C with mouse anti-human CD66b (Biolegend), mouse anti-human CD56 (Biolegend), rabbit anti-human GS GM1, rabbit anti-human NCR1, rabbit anti-human myeloperoxidase (Abcam), rabbit anti-human citrulline histone H3 (Abcam), or rabbit anti-Carbonic Anhydrase 9 (Abcam). Slides were washed and then incubated for 2 h at room temperature with the following secondary antibodies: Alexa Fluor 555–coupled goat antibody to rabbit IgG (Abcam), Alexa Fluor 488–coupled goat antibody to rabbit IgG (Abcam), or Alexa Fluor 488–coupled goat antibody to mouse IgG (Abcam). Slides were then washed and counterstained with DAPI before taking images using a fluorescence microscope (Leica). To quantitatively calculate the number of tissue-infiltrating cells, we count 5 different immunofluorescence staining fields of 1 sample and calculate the mean number of cells per field.

**Filipin III staining**

NK cells or neutrophils were fixed with 4% paraformaldehyde (PFA) and stained in Filipin III (50 μg ml^-1^) for 30 min. After washing three times with PBS, images were taken using a confocal laser scanning microscope (Leica), and the mean fluorescence intensity was determined using ImageJ.

**Modulation of cholesterol level**

To reduce the cholesterol level within DNT NK or neutrophils, 1 or 5 mM MβCD (Sigma) was used to treat cells for 5 min at 37 °C. Equal volume of PBS was added as negative control.

To add cholesterol to Tumor NK, NK cells were incubated with the culture medium supplied with MβCD-coated cholesterol (Sigma, 10 or 20 μg ml^-1^) at 37 °C for 15 min. Equal volume of PBS was added as negative control.

**Real-time PCR**

Total RNA was isolated from indicated cells by RNA extraction using the TRIzol reagent (Invitrogen). After RNA purification and concentration detection, total RNA was reverse transcribed into complementary DNA (cDNA) using the Hifair^®^ Ⅱ 1st Strand cDNA Synthesis SuperMix for qPCR kit (Yeasen). Real-time PCR analyses were performed using a LightCycler^®^ 96 (Roche) with SYBR green as a fluorescence marker (qPCR SYBR Green Master Mix, Yeasen) using sequence specific primers. Quantification was performed by normalizing to standard curves for each gene. The target genes were presented as their ratio to Ribosomal 18S RNA. The primer sequences used for RT-PCR was listed in **Table S1**.

**Western blot**

Cells were lysed in Triton lysis buffer (20 mM Tris, pH 7.4, 137 mM NaCl, 10% glycerol, 1% Triton X-100, 2 mM EDTA, 1 mM PMSF, 10 mM sodium fluoride, 5 mg ml^-1^ of aprotinin, 20 mM leupeptin and 1 mM sodium orthovanadate) and centrifuged at 12,000 g for 15 min. Sample supernatants were removed and protein concentrations were determined using the BCA assay kit (Invitrogen) according to the manufacturer’s instructions. 5×SDS-PAGE loading buffer (2 ml 1M Tris-HCl pH 6.8, 0.8g sodium dodecyl sulfate, bromophenol blue, 4 ml glycerol and 0.617g dithiothreitol) was added to the samples based on the protein concentrations. After heating at 95°C for 10 min, lysates containing equal amounts of protein were separated by SDS/PAGE and transferred to PVDF membranes. The proteins were then blocked for 1 h at room temperature in 5% BSA. Membranes were incubated in primary antibody overnight at 4 °C. The specific primary antibodies used were: anti-NCR1, anti-NKG2D, anti-p-ZAP70, anti-p-VAV (Abcam); anti-Caveolin-1, anti-Actin (Cell Signaling Technology); anti-CD36 (Abcam); anti-LRP1 (Abcam); anti-TAK1 (Cell Signaling Technology); anti-phosphorylated-TAK1 (Cell Signaling Technology); anti-IκBα (Cell Signaling Technology); anti-phosphorylated-IκBα (Cell Signaling Technology), anti-GAPDH (Abcam), anti-H3K36me3 (Cell Signaling Technology), anti-H3K27me3 (Abcam), anti-H3 (Abcam). Membranes were subsequently washed and incubated in appropriate fluorescein-conjugated secondary antibody, and then detected using a Tanon 4200F fluorescence scanner.

**Cytotoxicity measurement**

NK cells were cultured in complete RPMI-1640 medium after isolation. Human NK cells obtained from peripheral blood were stimulated with activation cocktail and then used for cytotoxicity measurement by cytotoxicity LDH assay kit (Dojindo Molecular Technologies). Briefly, target cells (K562 cells) were washed in PBS and plated out in round bottom 96-well plates (4×10^3^ cells per well) with NK cells in 100 μl medium (phenol-free RPMI 1640, 2% FBS) at indicated effector:target (E:T) ratio. High background control (100 μl medium + 10 μl lysis buffer, B), high control (100 μl medium + target cells + 10 μl lysis buffer, C), low background control (only 100 μl medium, D), low control (100 μl medium + target cells, E) and test background control (100 μl medium + effector cells, F) were set to quantify the release of LDH of target cells. The plates were subsequently incubated at 37 °C for 4 h in a CO_2_ incubator, followed by addition of Working Solution and Stop Solution. For quantification of NK-mediated killing of target cells, the release of LDH from target cells was detected by measuring the absorbance at 490 nm using a microplate reader (BioTek).

**Lipid rafts imaging and isolation**

Alexa Fluor 488-conjugate Cholera toxin subunit B (CTB, Invitrogen) and caveolin-1 antibody (Cell Signaling Technology) were used to stain ganglioside GM1 and caveolin-1 on cell plasma membrane. For ganglioside GM1 staining, cells were resuspended in medium (RPMI 1640, 10% FBS) containing Alexa Fluor 488-conjugate CTB (1 μg ml^-1^) at 4 °C for 10 min. After washing with PBS, the cells were further stained with DAPI and fixed with 4% PFA for 15 min. For caveolin-1 staining, cells were incubated with anti-caveolin-1 antibody (D46G3) at 1:400 dilution in 100 µl PBS at 4 °C for 30 min, followed by the Alexa Fluor 488–coupled goat antibody to rabbit IgG (Abcam) at 1:500 dilution in PBS (100 µl) at 4 °C for 30 min. After washing with PBS, the cells were stained with DAPI and fixed with 4% PFA for 15 min. The mean intensity of fluorescence was quantified using flow cytometry. In experiments testing co-localization of lipid rafts and activating receptors, cells were further incubated with rabbit anti-human NCR1 or rabbit anti-human NKG2D antibody (both from Abcam) for 30 min before PFA fixation. Images were taken using a confocal laser scanning microscope (Leica), in which co-localization was represented in yellow. Quantification of co-localizations was performed using ImageJ software, and presented as the percentage of NCR1 or NKG2D puncta that co-localized with CTB.

Lipid rafts proteins were extracted using the Ready Prep Protein Extraction Kit (Bio-Rad) following the manufacturer’s instructions. Briefly, 1×10^6^ cells were suspended in 150 µl buffer S1 and then sonicated on ice with an ultrasonic probe to break open the cells and fragment the genomic DNA. Then, 150 µl chilled buffer S2 was added into the cell extract. After vortexing 4-5 times, the tube was incubated in an ice-water bath for 15 min. After centrifugation at 15,000 g for 20 min at 4°C, the supernatant was collected as non-lipid rafts protein, and the pellet was resuspended in 50 µl buffer S1. The above steps were repeated to remove the remaining non-lipid rafts protein. The final pellet containing the lipid rafts proteins were resuspended in 60 µl complete PSB, and then centrifugated at 15,000 g for 10 min at room temperature. The supernatant was collected as lipid rafts proteins. The concentrations of proteins were determined by the RC DC Protein Assay kit (Bio-Rad).

**RNA sequencing**

CD66b^+^CD16^−^ neutrophils (from DNTs) and CD66b^+^CD16^+^ neutrophils (from CRC tumors) were sorted by FACS, and RNA sequencing (RNA-seq) was performed on these sorted neutrophils. Qubit 2.0 (Life Technologies) was used to analyze the RNA quality and integrity. A total of RNA (5 μg) was used for the RNA sample preparations. Extracted RNA samples were processed using the NEBNext^®^ UltraTM RNA Library Prep Kit (Lexogen) and sequenced on an Illumina Hiseq X-Ten with control of Hiseq Control Software (HCS). The sequencing library was qualified by Qubit 2.0 (Life technologies) and Bioanalyzer 2100 (Agilent). Raw reads were processed through in-house perlscripts. Clean reads were obtained by removing reads containing adapter. Differentially expressed genes were defined by P < 0.05 and an absolute fold change > 2. Using Gene Set Enrichment Analysis (GSEA), enrichment of a specific gene set was tested, and core enrichment genes were determined.

**Isolation and purification of NETs**

NETs were isolated from peripheral neutrophils. Briefly, neutrophils were cultured in RPMI-1640 medium with 500 nM phorbol myristate acetate (PMA) for 4 h to release NETs. After the supernatant was removed, the NETs adhered at the bottom were washed down by pipetting 2 ml cold PBS and centrifuging at 1,000 g for 10 min at 4 °C, and the cell-free supernatant with NETs was collected. The DNA concentration of NETs was measured by spectrophotometry and the NETs were used for further experiments.

**Chromatin immunoprecipitation (ChIP)**

The collected chromatin DNA was sheared into 200-500 bp fragments with ChIP Assay Kit (Beyotime) following the manual. Samples were then incubated with anti-H3K36me3 antibodies overnight at 4 ℃. Protein A/G plus agarose beads were added into the samples and incubated at 4 ℃ for 4 hours. Samples with beads were washed with ChIP wash buffers, and purified with DNA Purification Kit (Beyotime) to obtain DNA fragments. DNA fragments were stored at -80 ℃ for the following analysis. The primer sequences used for quantification are listed in **Table S1**.

**Quantification of cholesterol**

The cholesterol level of cell lysate was quantified using Amplex Red Cholesterol Assay (Invitrogen). Experiment was processed following the protocol on the manufacturer’s instructions. Briefly, cholesterol-containing samples were diluted in the 1X Reaction buffer and mixed with working solution which containing HRP (2 U ml^-1^), cholesterol oxidase (2 U ml^-1^) and cholesterol esterase (0.2 U ml^-1^) . Then this mixture was incubated for 30 minutes at 37 ℃, and cholesterol concentration was determined by comparing the 590 nm fluorescence emission to the standard curve.

**Cell stimulation**

For the activation of NK cells, NK cells including tumor NK, DNT NK and NK cells were all stimulated with IL-2 (20 ng ml^-1^) and IL-15 (20 ng ml^-1^) (leukocyte activation cocktail) for 12 hours before flow cytometry analysis or co-culture with organoids and other cells.

For the stimulation of neutrophils, CD16^+/-^ neutrophils were stimulated with human IgG (1 μg ml^-1^) for 6 hours, and neutrophils treated with equal volume of PBS were set as negative control. For the blockage of TAK1, CD16^+/-^ neutrophils were treated with 5z-7-oxozeaenol (20 nM) or equal volume of DMSO for 30 min on ice, then washed 3 times with PBS and stimulated with IgG.

**Extracellular fluid isolation**

Extracellular fluid (EF) isolation was proceeded as previously described.^[1]^ Briefly, CRC tissues were collected, dissected and placed into 20 μm nylon meshes (Millipore Sigma), and fixed in a 15 ml tube. These combined devices were subsequently centrifuged at 600g for 10min. EF was then frozen and kept at -80 ℃ for further analysis.

**Cell viability detection**

For the viability detection of HT29/HCT116 in the co-culture system, the HT29/HCT116 were resuspended in 500 μl medium per well, and then cells were seeded into a 96-well plate with 100 μl in a single well. After cultured at 37 ℃, 5% CO_2_ overnight, the CCK-8 kit (10 μl per well) was added and incubated at 37 ℃, 5% CO2 for 4 hours, then 450 nm OD value was detected with a microplate reader.

For the viability detection of PDO and dHL-60 in the PDO co-culture system, suspended dHL-60 was sucked out, and organoids were digested and resuspended. Then tumor cells or dHL-60 were seeded into 96-well plates. Each plate was placed at 37 ℃, 5% CO_2_ overnight and followed by CCK8 detection.

**Fluorescence intensity quantification**

Mean fluorescence intensity (MFI) was assessed by ImageJ 1.53e. Generally, RGB images s were split into 3 channels, and the threshold of the channel to be analyzed was automatically set by the software. To eliminate the interference of background signal and the different number of cells, the MFI was only calculated in the image local where the signal strength exceeds the threshold value. For the MFI quantification of organoids, cell trace dye (red) was used to label the region of organoids, and then the MFI of caspase 3/7 (green) in the cell-trace-dye-labeled region was calculated as described above.

**References in supplementary materials and methods**

1. M. J. Mittenbuhler, M. P. Jedrychowski, J. G. Van Vranken, H. G. Sprenger, S. Wilensky, P. A. Dumesic, Y. Sun, A. Tartaglia, D. Bogoslavski, M. A, H. Xiao, K. A. Blackmore, A. Reddy, S. P. Gygi, E. T. Chouchani and B. M. Spiegelman. Isolation of extracellular fluids reveals novel secreted bioactive proteins from muscle and fat tissues. *Cell Metab*, **2023**, *35*, 3.

**Figure S1
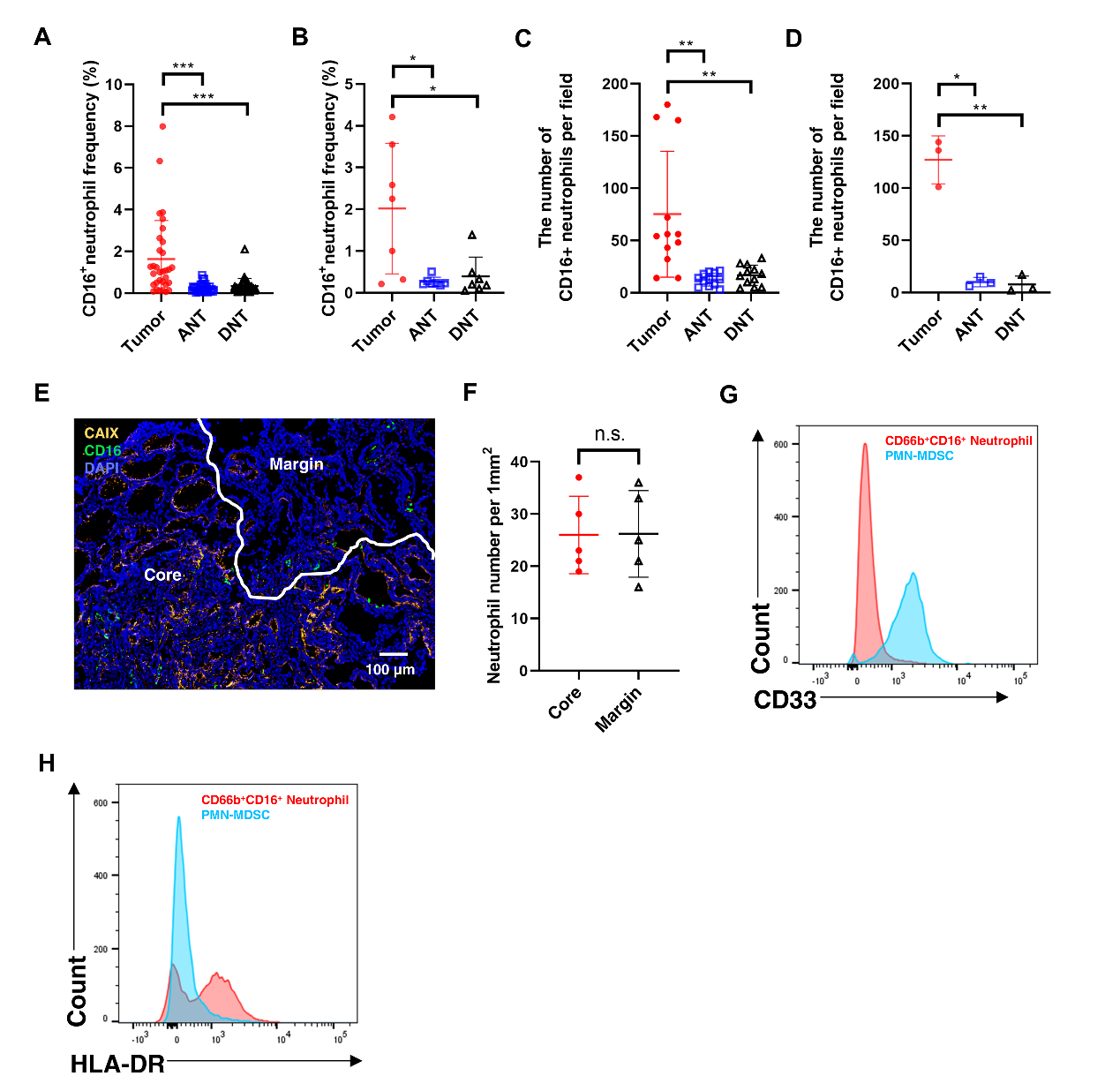
**

**Figure S1. CD16^+^ neutrophils were enriched in CRC tumor tissues.**

**A.** CD66b^+^CD16^+^ cell frequency from MSS CRC patients in (**Fig 1F**) (n=32). **B.** CD66b^+^CD16^+^ cell frequency from MSI CRC patients in (**Fig 1F**) (n=7). **C.** The number of neutrophils from MSS CRC patients in (**Fig 1H**) (n=12). **D.** The number of neutrophils from MSI CRC patients in (**Fig 1H**) (n=3). **E.** Representative images of immunofluorescence microscopy of CRC tumor tissue showing CD16^+^ cells (green) and CAIX (yellow). **F.** Quantification of the number of CD16^+^ neutrophils per 1 mm^2^ in (**E**) (n=5). **G.** Histogram of distribution obtained by flow cytometry showing the expression of CD33 of CD66b^+^CD16^+^ neutrophils from tumor and PMN-MDSC derived from peripheral blood. **H.** Histogram of distribution obtained by flow cytometry showing the expression of HLA-DR of CD66b^+^CD16^+^ neutrophils from tumor and PMN-MDSC derived from peripheral blood. Mean±SEM, *P < 0.05, **P < 0.01, ***P < 0.001, by two-tail t tests.

**Figure S2**
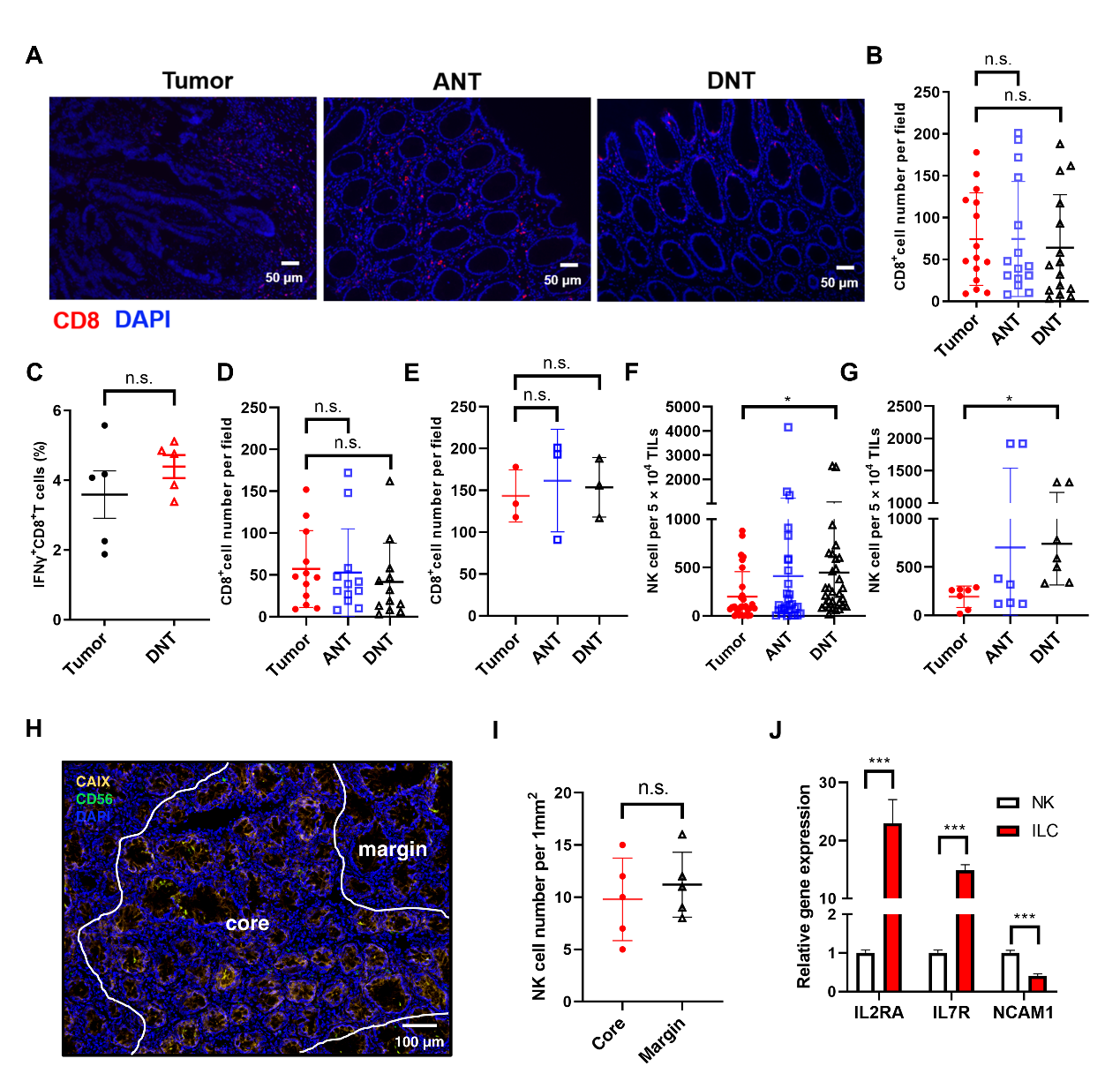


**Figure S2. Frequency of infiltrating CD8^+^ T cell showed no significant difference among CRC tumors, ANTs and DNTs.**

**A.** Representative images of immunofluorescence showing distributions of CD8^+^ (red) cells. **B.** Quantification of CD8^+^ T cell in tumor tissues, ANTs and DNTs (n=15). **C.** The percentage of IFN-γ^+^ CD8^+^ T cells in tumor tissues and DNTs (n=5). **D.** The number of CD8^+^ T cells from MSS CRC patients in (**B**) (n=12). **E.** The number of CD8^+^ T cells from MSI CRC patients in (**B**) (n=3). **F.** The number of NK cells from MSS CRC patients in (**Figure 2D**) (n=30). **G.** The number of NK cells from MSI CRC patients in (**Figure 2D**) (n=7). **H.** Representative images of immunofluorescence microscopy of CRC tumor tissue showing distribution of CD56^+^ NK cells (green) and CAIX (yellow). **I.** Quantification of the number of CD56^+^ NK cells per 1 mm^2^ in (**H**) (n=5). **J.** Relative mRNA levels of CD127 (*IL7R*), CD25 (*IL2RA*), and CD56 (*NCAM1*) in CD56^+^ NK cells and Lin^-^CD25^+^CD127^+^ ILCs (Lin: CD3, CD11b, CD19, CD14, CD66b, CD16). Mean±SEM, *P < 0.05, ***P < 0.001, by two-tail *t* tests.

**Figure S3**
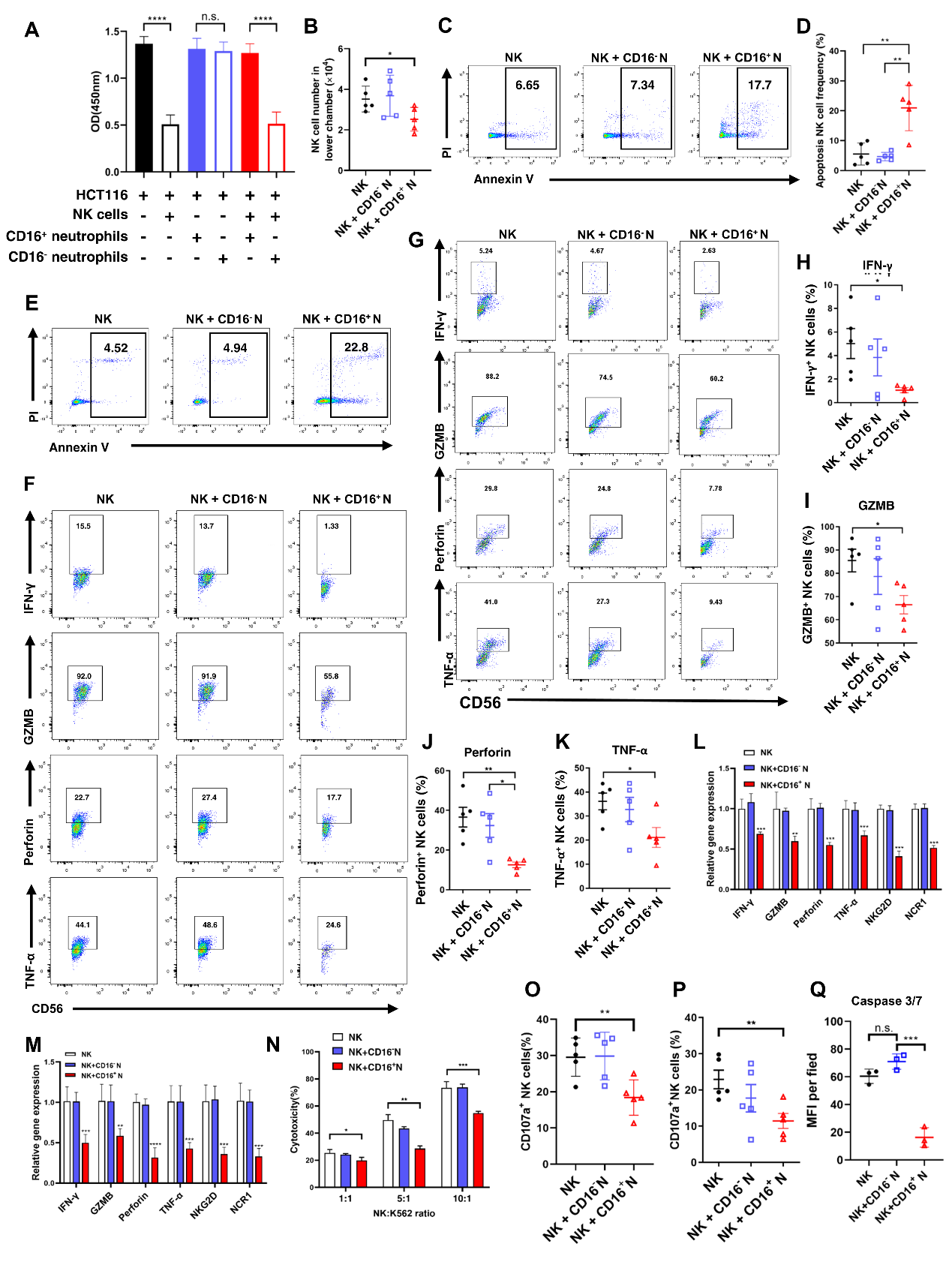


**Figure S3. CD16^+^ neutrophils were involved in the dysfunction of NK cells in CRC.**

**A.** Viability of HCT116 cells in the co-cultures as in **(Ai)** assessed by CCK8 assay (n = 6 per group). **B.** Quantification of the number of NK cells in the lower chamber of the co-culture system containing HCT116 in **(Ai). C.** Representative dot plot of flow cytometry and quantification showing the percentages of apoptosis NK cells (Annexin V^+^PI^+^) in the co-culture system containing HT29. **D.** Quantification of the frequency of NK cells undergoing apoptosis (Annexin V^+^PI^+^) in the co-culture system containing HCT116. **E.** Representative dot plot of flow cytometry and quantification showing the percentages of apoptosis NK cells (Annexin V^+^PI^+^) in the co-culture system containing HCT116. **F.** Representative dot plot showing frequencies of TNF-α^+^, Perforin^+^, GZMB^+^, IFN-γ^+^ cells in NK cells in the co-culture system containing HT29. **G.** Representative dot plot showing frequencies of TNF-α^+^, Perforin^+^, GZMB^+^, IFN-γ^+^ cells in NK cells in the co-culture system containing HCT116. **H to K.** Quantification of IFN-γ^+^ (**H**), GZMB^+^ (**I**), Perforin^+^ (**J**), TNF-α^+^ (**K**) NK cells among NK cells in the co-culture system containing HCT116. **L and M.** Relative mRNA levels of anti-tumor related pro-inflammatory gene expression in NK cells after co-culture with CD16^-^ neutrophils or CD16^+^ neutrophils in the co-culture system containing HT29 (**L**) or HCT116 (**M**). **N.** Quantification of cytotoxicity of NK cells in the co-culture system containing HCT116. **O and P.** in the co-culture system containing HT29 (**O**) or HCT116 (**P**). **Q.** Quantification of mean fluorescence intensity (MFI) of Caspase3/7 assessed by ImageJ in (**Figure 3J**, green) (n = 3). Mean±SEM, **P < 0.01, ***P < 0.001, ****P＜0.0001, by two-tail *t* tests.

**Figure S4**
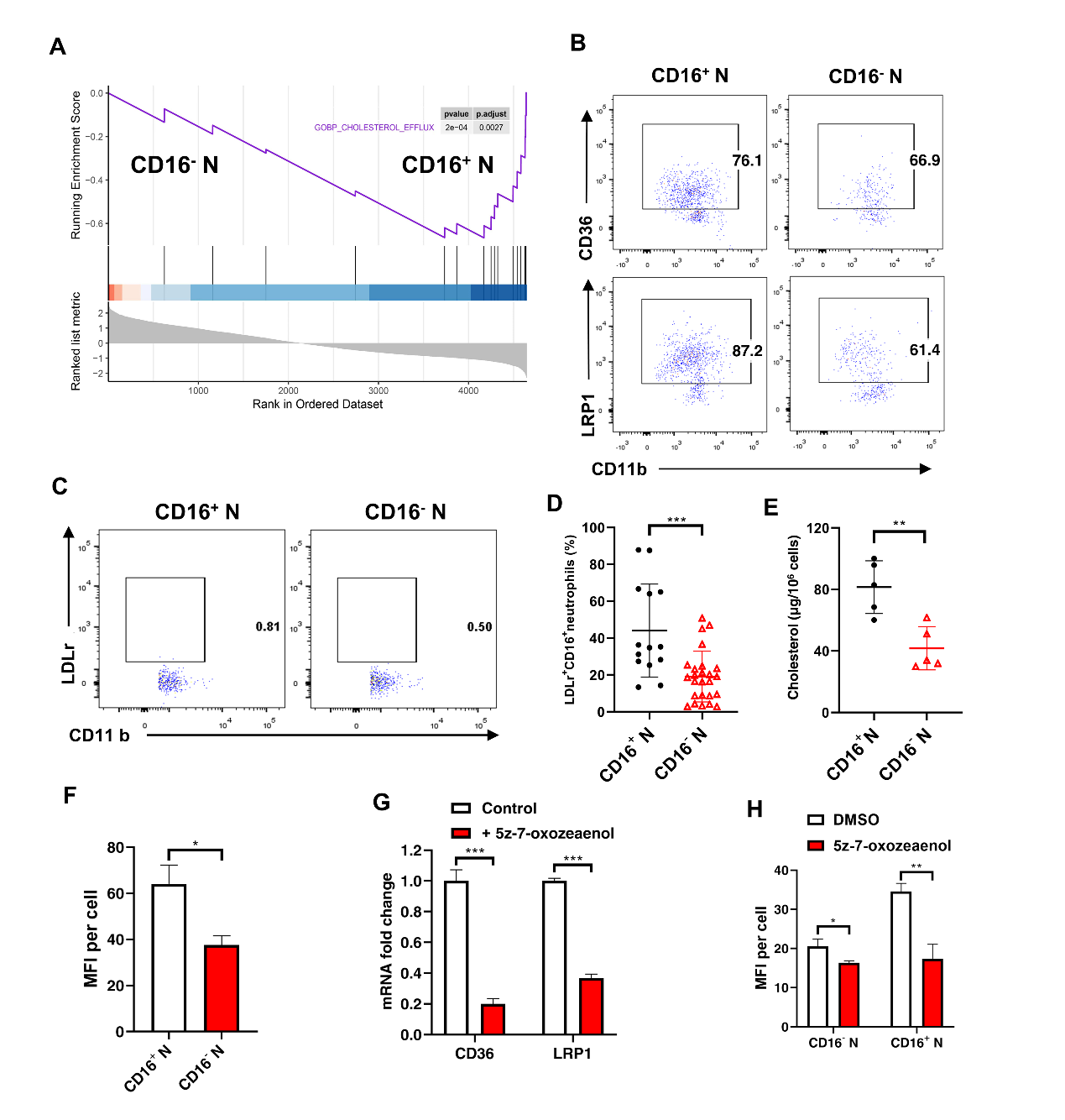


**Figure S4. Cholesterol efflux pathway was upregulated in CD16^+^ neutrophils and the expression of LDLR in neutrophils.**

**A.** Proteomic GSEA enrichment plots of Cholesterol Efflux in CD16^+^ neutrophils versus CD16^-^ neutrophils. **B.** Representative dot plot of flow cytometry showing percentages of LRP1^+^ and CD36^+^ cells in CD16^+^ and CD16^-^ neutrophils. **C.** Representative dot plot of flow cytometry showing percentages of LDLR^+^ cells in CD16^+^ and CD16^-^ neutrophils. **D.** Quantification of frequencies of LDLR^+^ cells in (**C**). **E.** Cholesterol quantification of CD16^+^ and CD16^-^ neutrophils. **F.** Quantification assessed by ImageJ of mean fluorescence intensity in (**Figure 4G**). **G.** Relative expression of CD36 and LRP1 in CD16^+^ neutrophils with or without 5z-7-oxozeaenol. **H.** Quantification assessed by ImageJ of fluorescence intensity in (**Figure 4J**). Mean±SEM, *P < 0.05, **P < 0.01, ***P＜0.001, by two-tail *t* tests.

**Figure S5**
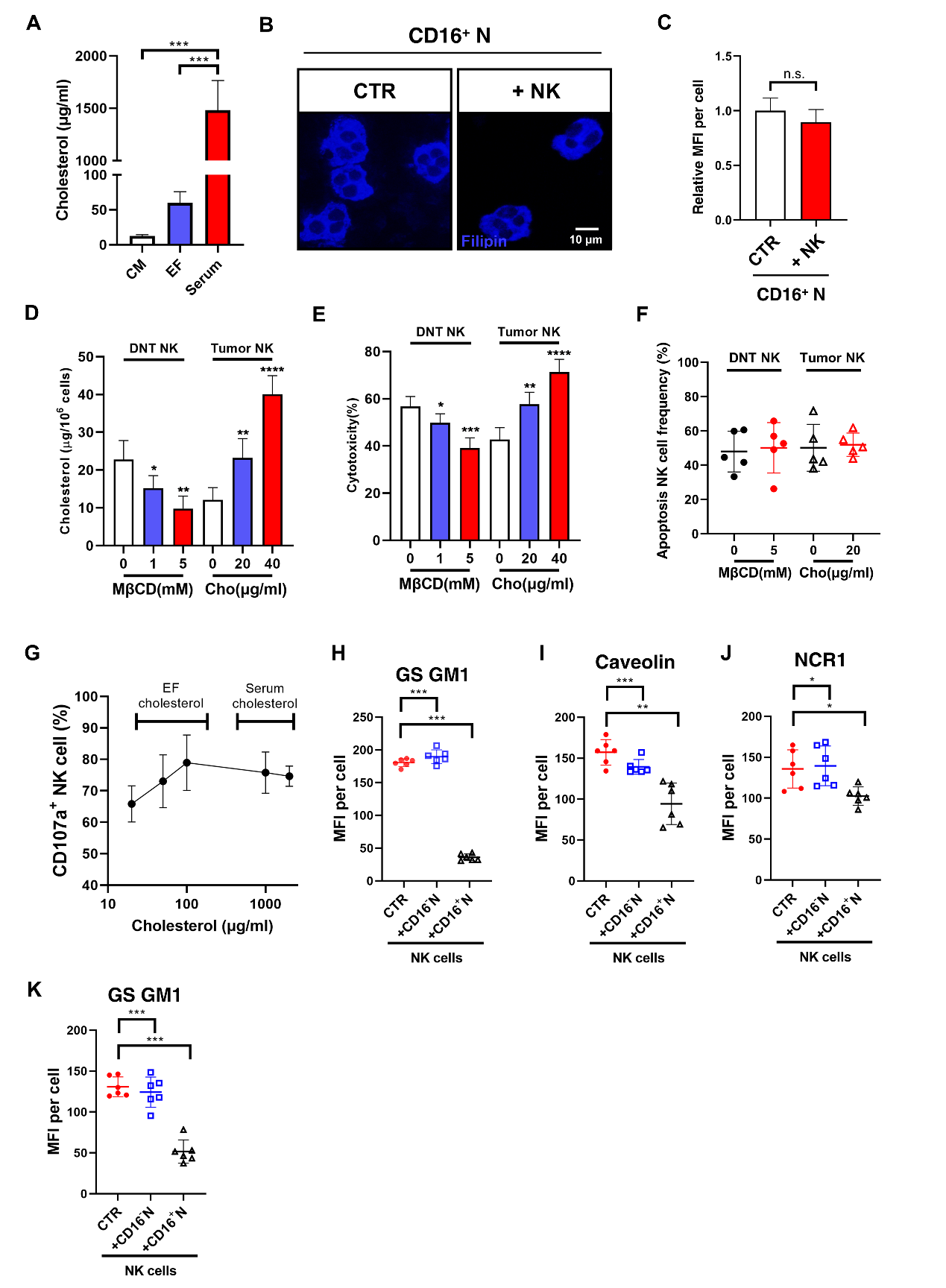


**Figure S5. The cholesterol level in NK cells.**

**A.** Cholesterol concentration in culture media (CM), tissue extracellular fluid (EF) and serum. **B.** Filipin III staining of CD16^+^ neutrophils with or without NK cells. **C.** Quantification of mean fluorescence intensity in (**B**). **D.** Cholesterol quantification of normal NK cells cultured in complete RPMI 1640 medium with 0, 1 or 5 mM MβCD, and tumor NK cells with 0, 20 or 50 μg/mL exogeneous cholesterol. **E.** Quantification of cytotoxicity of DNT NK cells cultured in complete RPMI 1640 medium with 0, 1 or 5 mM MβCD, and Tumor NK cells with 0, 20 or 50 μg/mL exogeneous cholesterol. **F.** Quantification of the frequency of apoptosis NK cells (Annexin V^+^PI^+^). **G.** The line chart showing the proportion of CD107^+^ cells in stimulated CD56^+^ NK cells after cultured with different concentration of exogeneous cholesterol. **H and I**. Quantification of mean fluorescence intensity per cell of GS GM1 (**H**) and Caveolin (**I**) assessed by ImageJ in (**Figure 5M**) (n = 6). **J and K**. Quantification of mean fluorescence intensity per cell of NCR1 (**J**) and GS GM1 (**K**) assessed by ImageJ in (**Figure 5N**) (n = 6). Mean±SEM, *P < 0.05, **P < 0.01, ***P < 0.001, ****P＜0.0001, by two-tail *t* tests.


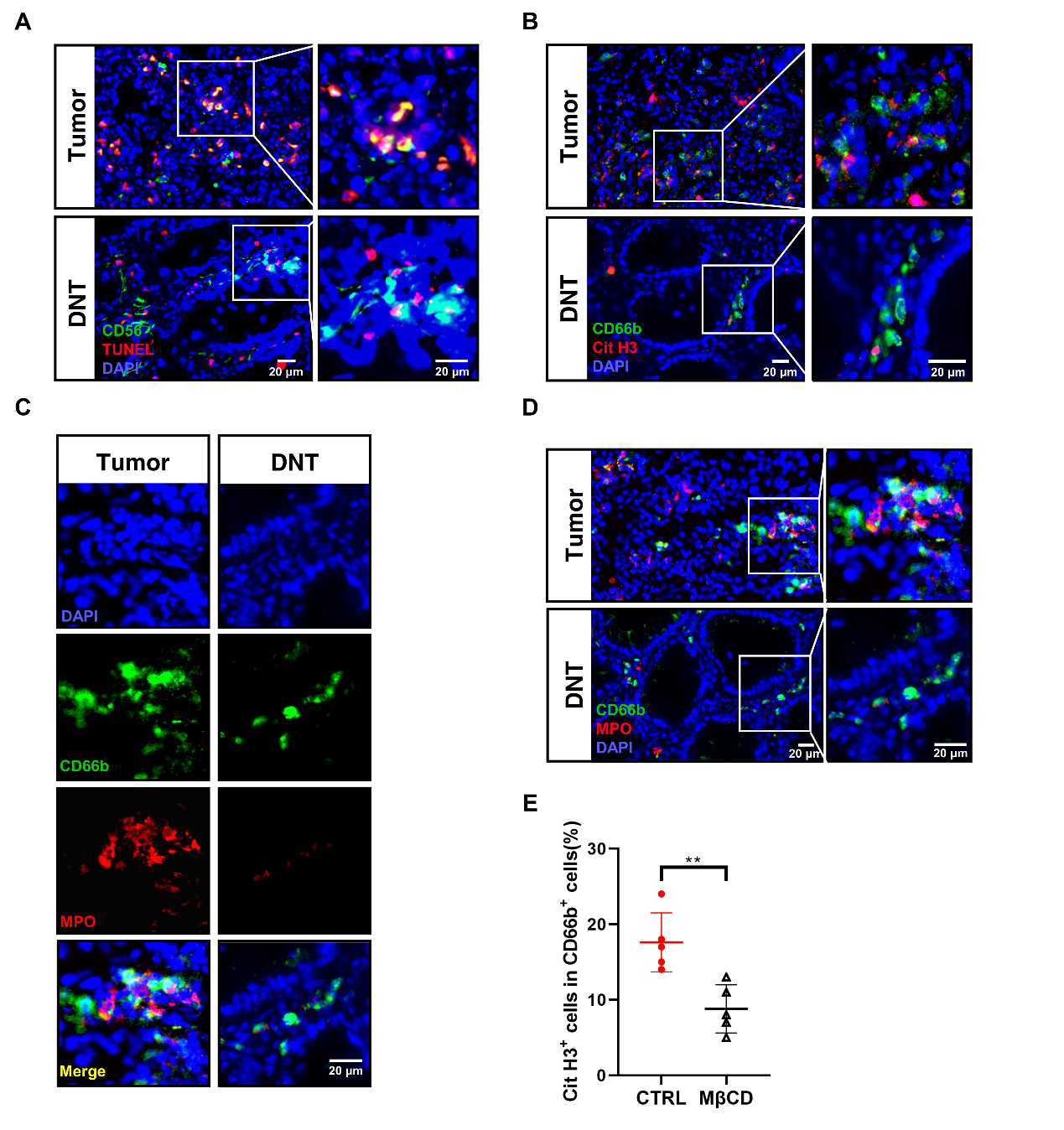
**Figure S6**

**Figure S6.**

**A.** Representative images of immunofluorescence microscopy of CRC tumor tissue and DNT showing CD56^+^ cells (green) and TUNEL (TdT-mediated dUTP Nick-End Labeling, red) at low magnification, and the selected areas by white frames were shown at high magnification. **B.** Representative images of immunofluorescence microscopy of tumor and DNT showing CD66b^+^ cells (green) and Cit H3 (red) at low magnification, and the selected areas by white frames were shown at high magnification. **C and D.** Representative images of immunofluorescence microscopy of tumor and DNT showing CD66b^+^ cells (green) and MPO (red). **E.** Quantification of the proportion of Cit H3^+^ cells in CD66b^+^ cells in (**Figure 6I**). Mean±SEM, **P < 0.01.


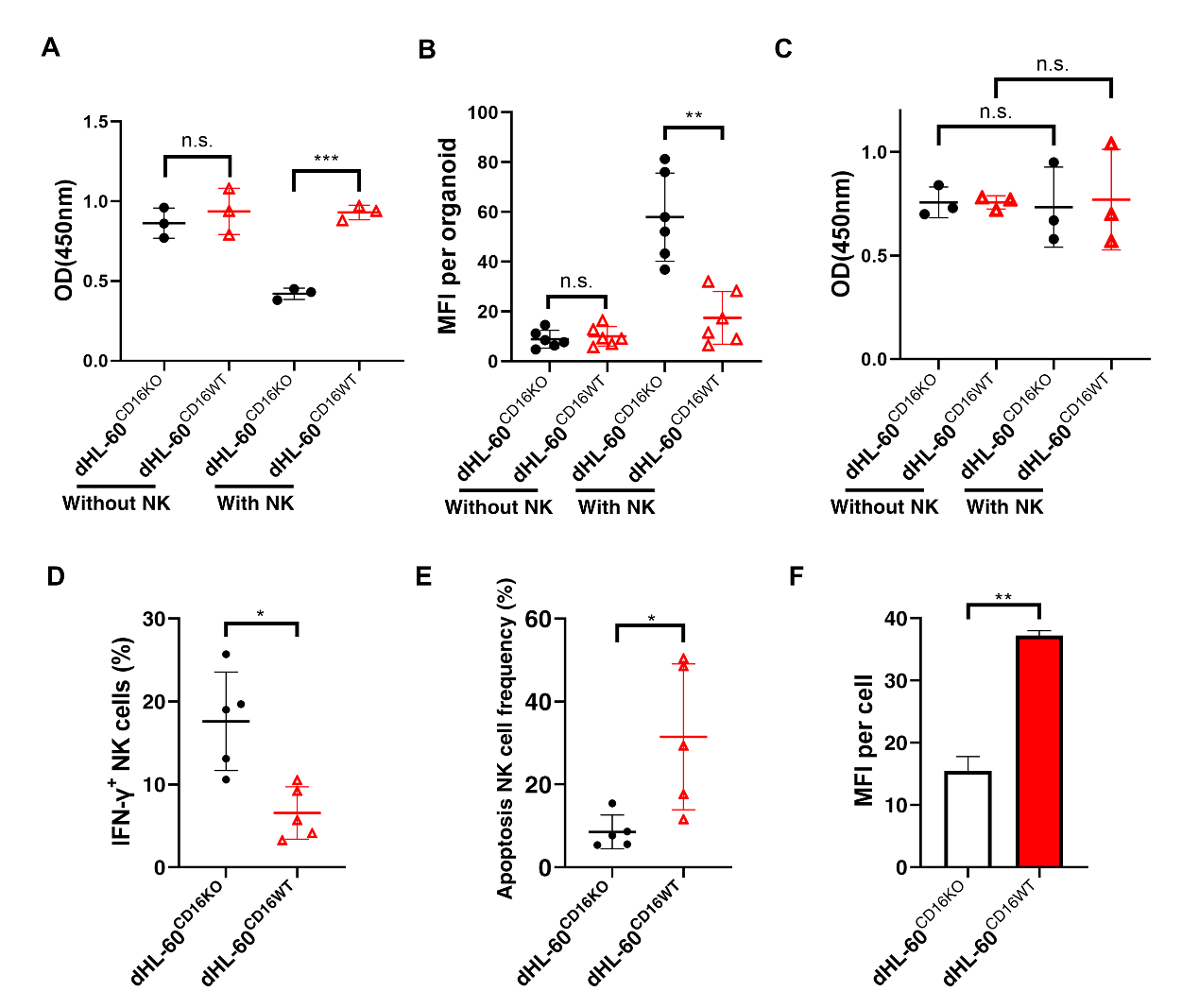
**Figure S7**

**Figure S7.**

**A.** Viability of CRC PDO in the co-cultures as in (**Figure 7B**) assessed by CCK8 assay (n = 3 per group). **B.** Quantification of mean fluorescence intensity per organoid of Caspase 3/7 in (**Figure 7B**) (n = 6 per group). **C.** Viability of dHL-60 in the co-cultures as in (**Figure 7B**) assessed by CCK8 assay (n = 3 per group). dHL-60 was separated from the co-culture system by magnetic sorting before analysis. **D.** Quantification of the percentages of IFN-γ^+^ NK cells in (**Figure 7C**). **E.** Quantification of the percentages of apoptotic NK cells in (**Figure 7D**). **F.** Quantification of mean fluorescence intensity in (**Figure 7F**). Mean±SEM, *P < 0.05, **P＜0.01, ***P < 0.001, by two-tail *t* tests.

**Table S1. Reagent sequences**

| **Reagent** | **Sequence (5' to 3')** | **Source** |
| --- | --- | --- |
| IFNG primer-fwd | GCCACGGCACAGTCATTGA | Qin WH et al. Gastroenterology. 2020 |
| IFNG primer-rev | TGCTGATGGCCTGATTGTCTT | Qin WH et al. Gastroenterology. 2020 |
| GZMB primer-fwd | CCACTCTCGACCCTACATGG | Qin WH et al. Gastroenterology. 2020 |
| GZMB primer-rev | GGCCCCCAAAGTGACATTTATT | Qin WH et al. Gastroenterology. 2020 |
| PRF primer-fwd | TCCACAGAGCATGCTTACCA | Qin WH et al. Gastroenterology. 2020 |
| PRF primer-rev | ACGGTAGGTCTGGTGGAAAG | Qin WH et al. Gastroenterology. 2020 |
| TNFa primer-fwd | AAGCCTGTAGCCCACGTCGTA | Qin WH et al. Gastroenterology. 2020 |
| TNFa primer-rev | GGCACCACTAGTTGGTTGTCTTTG | Qin WH et al. Gastroenterology. 2020 |
| CD36 mRNA primer-fwd | AACCACACACTGGGATCTGAC | This paper |
| CD36 mRNA primer-rev | CTGCAGGAAAGTCCTACACTG | This paper |
| LRP1 mRNA primer-fwd | GCCTCCTACCACTTCCAACC | This paper |
| LRP1 mRNA primer-rev | TTCATCTGCGCCACCTCAAT | This paper |
| IL2RA mRNA primer-fwd | AAGAGGCCCCAGGCAAGTA | This paper |
| IL2RA mRNA primer-rev | TTTGGAGAGGAGCTTGGTTGT | This paper |
| IL7R mRNA primer-fwd | GGGTCCCTCCTAAGACCCTA | This paper |
| IL7R mRNA primer-rev | GCCAGCATGTTCCTCAATGG | This paper |
| NCAM1 mRNA primer-fwd | TACGTGGACTGCTAAGGCTG | This paper |
| NCAM1 mRNA primer-rev | AGAATTTTCCAGGGAGGCCG | This paper |
| CD36 p50 binding site primer-fwd | GAAATCTCCGTGGCTGGAGT | This paper |
| CD36 p50 binding site primer-rev | CACCCTTTGAGCAAGTCCAA | This paper |
| LRP1 p50 binding site primer-fwd | GAAATCTCCGTGGCTGGAGT | This paper |
| LRP1 p50 binding site primer-rev | CGGTATCTGGAGCAACCACA | This paper |
| FCGR3B_gRNA1 | TCAAGCACGCTGTACCATTG | addgene library |
| FCGR3B_gRNA2 | GCTGCCACAGTCAACGACAG | addgene library |
| NonTargetingControl | AGTTCCCAGAAATATATTGC | addgene library |
